# Assessment of Aerobic Fitness and Repeated Sprint Ability in Elite Male Soccer: A Systematic Review of Test Protocols Used in Practice and Research

**DOI:** 10.1007/s40279-025-02188-4

**Published:** 2025-04-12

**Authors:** Nikolaos D. Asimakidis, Chris Bishop, Marco Beato, Anthony N. Turner

**Affiliations:** 1 Faculty of Science and Technology, London Sport Institute, Middlesex University, London, UK; 2 Performance Department, Ipswich Town Football Club, Ipswich, UK; 3 School of Health and Sports Sciences, University of Suffolk, Ipswich, UK

## Abstract

**Background:**

Soccer requires players to cover distances around 10–12 km, with numerous consecutive sprints throughout the 90-min game. As such, aerobic fitness and repeated sprint ability (RSA) are crucial physical qualities for the modern soccer player to cope with the demands of the game. However, a comprehensive and systematic search of aerobic fitness and RSA assessment procedures in elite soccer has yet to be conducted.

**Objectives:**

The aims of this systematic review were to (1) identify the tests and outcome variables used to assess aerobic fitness and RSA of elite male soccer players, (2) provide normative values for the most common tests of aerobic fitness and RSA across different playing levels, and (3) report the reliability values of these aerobic fitness and RSA tests.

**Methods:**

A systematic review of the academic databases MEDLINE, CINAHL, SPORTDiscus, Web of Science, and OVID for studies published until August 2023 was conducted, following the Preferred Reporting Items for Systematic Reviews and Meta-Analyses (PRISMA) guidelines. Studies were eligible for inclusion if (1) they were original research studies, published in a peer-reviewed journal, and written in English language; (2) they had the primary aim of assessing aerobic fitness and/or RSA; (3) players were male and older than 17 years of age (i.e. mean age of the group); and (4) their playing level was defined as ‘professional’, ‘international’, or ‘elite’.

**Results:**

For aerobic fitness testing, 124 studies and 35 different tests were identified. Of those, 26 tests (74%) were field-based, whereas only nine (26%) were laboratory-based tests. The incremental treadmill test to exhaustion was the most commonly used aerobic fitness assessment method (56 studies, 45%), with maximal oxygen consumption ($\dot{V}$O_2max_) (mL/kg/min) being the most prevalent outcome variable (49 studies, 87%). The YYIR1 and YYIR2 were also commonly used tests, identified in 22 (18%) and ten studies (8%), respectively. The most frequently reported outcome variable in both tests was distance in metres, reported in 20 studies (91%) for YYIR1 and in all ten studies (100%) for YYIR2. For RSA testing, 27 studies and 18 different tests were identified. Substantial variability in the identified RSA testing protocols was observed in terms of direction (linear vs. multidirectional), sprint repetitions (6–15), sprint distance (20–40 m), type of recovery (active vs. passive), and recovery duration (10–30 s). The 6 × 40-m shuttle sprint protocol with a 180° change of direction and 20 s passive recovery was the most common RSA test, employed in eight studies (29%).

**Conclusions:**

This systematic review provides a comprehensive overview of the testing methods used to assess aerobic fitness and RSA in elite male soccer players. A total of 35 different aerobic fitness tests and 18 RSA tests were identified, highlighting the diversity in methodologies used. The most prevalent aerobic test was the incremental treadmill testing to exhaustion, with a median $\dot{V}$O_2max_ value of 58 mL/kg/min. Field-based tests were preferred due to their practicality, cost-efficiency, and ability to assess multiple athletes simultaneously. A substantial variability in RSA testing protocols was identified in terms of sprint directions, distances, repetitions, and recovery types. Future research should focus on establishing the diagnostic accuracy of the most commonly used aerobic fitness tests to inform their utility in practice and bridge the gap between current testing practices and optimal fitness evaluation.

**Supplementary Information:**

The online version contains supplementary material available at 10.1007/s40279-025-02188-4.

## Key Points

**Table:** 

Assessing maximal oxygen consumption ($\dot{V}$O_2max_) was most commonly done using incremental treadmill testing to exhaustion, with elite soccer players demonstrating a median value of 58.2 mL/kg/min.
The majority of the identified aerobic tests were field based (27 out of 36), with the Yo-Yo intermittent recovery level 1 and 2 tests most commonly used.
The 30–15 Intermittent Fitness Test and submaximal fitness protocols were identified as emerging trends, with the 30–15 Intermittent Fitness Test offering increased specificity and enhanced prescriptive ability, and the submaximal fitness testing being less disruptive to the training process.
The relevance of most repeated sprint ability tests is questionable, since they do not replicate actual match-play demands or effectively target the physiological systems they aim to evaluate.
Reliability and sensitivity studies within the same athlete cohorts are needed to determine the diagnostic accuracy of the most commonly used tests. Additionally, the development of a soccer-specific Participant Classification Framework is required to address the ambiguity in defining ‘elite’ and ‘professional’ players.

## Introduction

Analyses of the physiological demands of soccer have revealed that elite soccer players run 10–12 km per game depending on playing position [1–3], mostly covered at low and moderate intensities [4]. Aerobic metabolism is the most prevalent source of energy during a game, contributing to a great extent of the total energy demands [5]. In particular, the average oxygen consumption ($\dot{V}$O_2_) during a match is around 70–80% of maximal oxygen consumption ($\dot{V}$O_2max_), with average heart rate (HR) typically corresponding to 80–90% of maximal values [6]. In addition, soccer players execute sprints at maximum or near-maximum speeds of short duration, typically lasting between 1 and 6 s, repeatedly throughout a 90-min game [7], with varying recovery periods in between [8, 9]. Previous research has shown a decline in running performance during the second half for both lower and higher intensity running [2, 10, 11]. Hence, a high aerobic capacity and the ability to recover quickly from successive sprints while maintaining maximal output during any subsequent sprints are essential qualities for effectively coping with the physical demands of the game [7, 12].

A higher level of aerobic capacity has been shown to be associated with a reduced injury risk [13] and a smaller fatigue-related deterioration of technical performance in soccer players (*r* = − 0.51 to − 0.65, *p* < 0.05) [14]. Furthermore, aerobic fitness can differentiate between level of play (i.e. professional vs. amateurs [15], sex [16], and age category) [17]. Differences exist in the aerobic performance of different playing positions, with midfielders generally exhibiting higher $\dot{V}$O_2max_ values [18, 19], underscoring the different aerobic demands of each position. Likewise, several studies have shown that repeated sprint ability (RSA), which refers to the ability to perform multiple short sprints with a minimal decrement in performance [20], represents a distinguishing factor between professional and amateur soccer players, as well as playing positions [21–23]. Studies examining the correlations between aerobic fitness and RSA in soccer players have elicited mixed findings [24–27]. However, a recent meta-analysis showed that correlations between aerobic fitness and RSA are low to moderate (*r* = 0.30–0.52, *p* < 0.05) [28], which may imply that they should be assessed separately.

Aerobic fitness and RSA testing selection presents a significant challenge for researchers and practitioners, given the wide variety of available testing protocols [29, 30]. Complementary to the physiological and biomechanical demands of soccer [31], test selection should also be based on the reliability and sensitivity of an instrument to facilitate an accurate representation of an individual's performance and progress over time [32]. Traditionally, the evaluation of $\dot{V}$O_2max_ with incremental exercise testing in a laboratory setting has been considered the gold-standard method of aerobic capacity assessment [33]. In addition to $\dot{V}$O_2max_, submaximal measures such as running economy, lactate threshold, and ventilatory threshold – obtained during incremental treadmill testing – enable the nuanced understanding of an athlete’s aerobic ability [34, 35]. These parameters are particularly relevant in soccer, where a great extent of the game is played at intensities below $\dot{V}$O_2max_ [7], and where improvements in these variables have been shown to occur even without improvement in $\dot{V}$O_2max_ [36]. However, laboratory-based assessments can be time-consuming and may fail to replicate the intermittent demands of field-based sports like soccer, thus reducing their ecological validity and specificity [30]. Besides greater specificity, field tests also offer the advantage of being easier to administer when working with large groups of athletes [30]. Furthermore, to address the logistical challenges of maximal testing in elite soccer, submaximal testing protocols have been suggested as a time-efficient and non-fatiguing alternative for evaluating aerobic adaptations [37]. On the other hand, the evaluation of RSA is performed through the implementation of sprints with various durations, distances, and directions (i.e. linear vs. shuttle) and includes the use of various measures, such as the best sprint time (RSA_best_), mean sprint time (RSA_mean_), total sprint time summed for all trials (RSA_total_), as well as indicators of fatigue (i.e. performance decrement and fatigue index) [38]. Notably, the neuromuscular and physiological load imposed on the players during RSA testing varies between tests that employ a different number of sprints, type, and amount of recovery [20].

Despite the well-established importance of aerobic fitness and RSA in soccer, a comprehensive synthesis of the testing protocols used at the elite level is missing. Soccer, especially at higher levels, is characterized by unique physiological demands compared to other team sports [39]. Moreover, distinct physiological and performance differences exist between male and female soccer players [2, 40]. While Bok and Foster [30] conducted a narrative review on the applicability of field aerobic fitness tests in soccer, offering valuable insights into test selection, an in-depth summary of the testing protocols used in scientific literature was not within the scope of their study. Previous systematic reviews have investigated either the testing protocols used to assess RSA [38] or the measurement properties of RSA tests [29, 41], although none of them have explicitly focused on the assessment methods employed in elite soccer. In addition, the investigation of normative values for both aerobic fitness and RSA is of high practical value, as it can supply practitioners with benchmarks to evaluate players’ fitness levels relative to elite-level standards. Previous research has shown differences in aerobic performances between regular first team players and elite youth players striving to make the transition to the first team [16, 17, 42]. By reporting normative values for both regular first team and elite youth players, a better contextualization of physical performance can be achieved, providing a clearer understanding of where the players stand relative to their competitive level. Having benchmark data like these facilitates a more strategic approach to athletic development, talent identification, and individualized training programming. Furthermore, examining the reliability of the identified tests and outcome variables is critical for determining the accuracy and the consistency of these measurement tools. Reliable tests provide practitioners with an increased confidence in the precision of the results, as well as in the interpretation of the performance changes over time [43]. By synthesizing the findings from multiple studies performed in high-level male soccer populations, this systematic review can serve as a valuable resource for practitioners and researchers alike, to understand current practices, historical trends, and areas requiring future investigation. Ultimately, the development of more robust and standardized testing practices can be promoted for both aerobic fitness and RSA. Consequently, the aims of this systematic review were to (1) identify the tests and outcome variables used to assess aerobic fitness and RSA of elite male soccer players, (2) provide normative values for the most common tests of aerobic fitness and RSA across different playing levels, and (3) report the reliability values of these aerobic fitness and RSA tests.

## Methods

### Design and Search Strategy

A systematic review was performed following the Preferred Reporting Items for Systematic Reviews and Meta-Analyses (PRISMA) statement [44]. The academic databases MEDLINE, CINAHL, SPORTDiscus, Web of Science, and OVID were searched from the earliest record (i.e. January 1982) to August 2023, to identify English-language, peer-reviewed, original research studies that evaluated aerobic fitness and/or RSA, in elite male soccer players. Key words used for the identification of the studies are shown in Table 1. Search levels 1–5 were all linked by the Boolean operator ‘AND’, whereas search terms within each search level were joined with ‘OR’ or ‘NOT’. All search results were extracted and imported into a reference management software (RefWorks, ProQuest LLC, Ann Arbor, Michigan, US).

**Table 1:** Search strategy terms

Search 1	Search 2	Search 3	Search 4	Search 5
Soccer OR Football NOT (‘American football’ OR ‘Australian Rules football’ OR rugby OR ‘Gaelic Football’)	Male OR men	Adult OR senior	Professional OR elite	Fitness testing OR physical characteristics OR testing OR physical performance OR physical qualities OR physical profile OR fitness OR physical fitness OR aerobic* OR repeated sprint ability

### Study Selection

Following the removal of duplicates, two reviewers (NA and CB) independently screened all titles and abstracts against the inclusion and exclusion criteria of the review. Studies that did not meet the inclusion criteria were removed. Any conflicts were addressed through discussion, or via the third reviewer (AT). The full texts of the articles that were included during this process were subsequently reviewed for eligibility. In addition to the systematic search, reference lists of the included papers were reviewed to identify potentially eligible articles. To fulfil the first objective of the review, studies were eligible for inclusion if they (1) were original research studies, published in a peer-reviewed journal, and written in the English language; (2) had the primary aim to assess aerobic fitness and/or RSA; (3) players were male and older than 17 years of age (i.e. mean age of the group), which was in line with a previous systematic review and a survey study on fitness testing [29, 45] and to minimize any potential influence of maturation [46]; and (4) their playing level was defined as ‘professional’, ‘international’, or ‘elite’. These playing levels correspond to tiers 3–5 (i.e. highly trained/national level, elite/international level, world class) of the Participant Classification Framework proposed by McKay et al. [47]. Conversely, studies were excluded from the review if they (1) were narrative or systematic reviews and/or meta-analyses; (2) assessed physical characteristics as a result of other research objectives (i.e. fatigue, recovery, nutrition, and genome); (3) the sample consisted of different team sports; (4) players were semi-professional; (5) players were younger than 17 years of age; or (6) reported tests included the use of a ball. For the second objective, studies were eligible if they reported the mean result of the tests under consideration and clearly distinguished between different groups (i.e. professional vs. amateurs, adult men vs. youth, male vs. female). As such, only normative data for elite male soccer players older than 17 years old were recorded. For the third objective, studies were included if they provided information about reliability statistics (i.e. within-day and/or between-day) of the sample used in the study and had a clear description of the procedures that had taken place.

### Assessment of Methodological Quality

The methodological quality of the included articles was assessed using a modified version of the Downs and Black [48] rating scale. This checklist has been used previously in systematic reviews with similar research objectives [49, 50] and can be adapted to the scope and the needs of the systematic review [51]. Eleven questions (1–4, 6, 7, 10, 11, 16, 18, 20) from the traditional version of the checklist were considered relevant to the specific aims of this systematic review, and therefore used to grade the methodological quality of the included studies (Supplementary Table S1 of the Electronic Supplementary Material [ESM]). For the purposes of this review, question 4 was directed to whether the testing procedures in each study were clearly described. Each question was scored as either a ‘1’ (yes) or a ‘0’ (no or unable to determine). Scores were summed for each study, with a total score of ‘11’ representing the highest possible score.

### Data Extraction

Data were extracted and documented in a Microsoft Excel 365 spreadsheet (Microsoft Corporation, Redmond, Washington, USA). Data extracted from each study consisted of the research design, publication details (authors and year of publication), sample information (number of participants, age of the sample, playing level), tests administered to assess aerobic fitness and/or RSA, outcome measures derived from each test, and normative values from each test. Where available, reliability values (i.e. intraclass correlation coefficient [ICC], coefficient of variation [CV], standard error of measurement [SEM], minimal detectable change [MDC], Pearson’s *r* and Cronbach’s alpha [*α*]) were also recorded. Playing level was divided into two categories: (1) senior professionals, representing players that were regular members of the first team of a professional soccer club and/or a national team’s senior squad, and (2) elite youth, which included players over 17 years old who were members of the youth department of a professional soccer club (but not yet regular members of the first team), were members of a junior national team squad, or were defined as ‘elite’ by the authors of the study. According to McKay et al.’s [47] Participant Classification Framework, senior professionals encompass athletes from tiers 3–5 (i.e. national level to world class), while elite youth players fall within tier 3 (i.e. national level/highly trained). In studies with more than one group of players, only the groups with a mean age of 17 years or older were included for subsequent analysis. In terms of reporting normative values, the mean of each group (i.e. senior professionals vs. elite youth) was recorded. For studies with multiple groups of the same playing level, the mean and pooled standard deviation were reported. In intervention studies, only the baseline values were recorded to eliminate any intervention bias – noting that the purpose of this review was not to undertake a meta-analysis evaluating training effects. When a repeated-measures, no intervention study design was implemented, such as seasonal variation studies, the most recent testing point was recorded, in line with a recent similar systematic review in rugby union players [49] and to capture the latest performance level, unless the most recent point was taken after a congested fixture or a detraining period.

## Results

### Identification and Selection of Articles

**Fig. 1:**
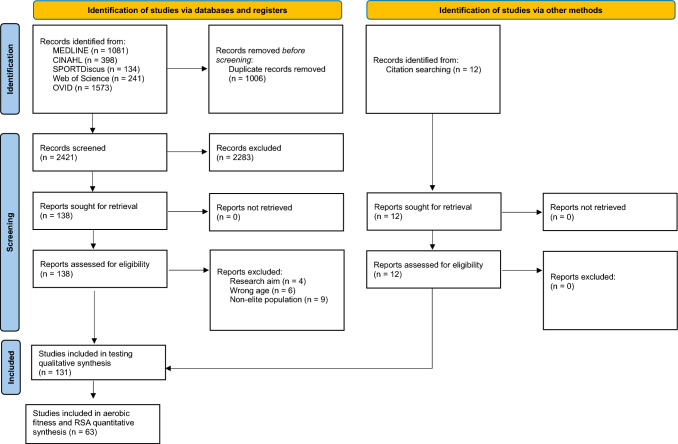
Flow of selection process of eligible studies for qualitative and quantitative synthesis

Figure 1 illustrates the flowchart of the selection process. The initial search of databases identified 3427 articles. After removing the duplicates (1006 articles), the titles and abstracts of 2421 articles were screened. A total of 138 articles were retained for eligibility assessment through full-text review. Twelve additional studies were identified through manually searching reference lists for full-text eligibility assessment. Following full-text screening, 131 were included for the aim of identifying the tests and outcome variables used to assess aerobic fitness and RSA in elite male soccer. In addition, 63 of those were included for the purpose of reporting normative values for the most common aerobic fitness and RSA tests, whereas only 19 studies reported reliability data.

### Characteristics of Included Studies

Supplementary Table S2 of the ESM shows the assessment of quality scores, with the 11 assessed items ranging from 6 to 10. Supplementary Table S3 of the ESM provides a summary of characteristics of the studies included in this systematic review. The range of sample sizes was 12–927, with a median of 24 participants. Ninety-two studies included senior professionals as participants, 29 included elite youth, while ten studies involved a group of both. The age range of the samples involved in the studies was 17.0–28.6 years, with a median age of 23.8 years. In terms of study design, 55 studies (42%) used a cross-sectional design, 47 (35.9%) used a repeated-measures design, 18 (13.7%) were intervention studies, seven (5.3%) were reliability studies, and six (4.6%) were validity studies. The studies took place in 32 different countries, with Spain (12.2%), Italy (8.4%), England (6.9%), Brazil (6.9%), and Greece (6.9%) being the most frequently identified countries.

### Tests and Outcome Variables Used to Assess Aerobic Fitness

Aerobic fitness was investigated in 124 studies (94.7%) (Tables 2 and 3). Thirty-five different tests were used to assess aerobic fitness, demonstrating the multitude of different assessment methods available. Of those, 26 tests (74%) were field-based, whereas only nine (26%) were laboratory-based tests. Incremental treadmill test to exhaustion was the most commonly used aerobic fitness assessment method (56 studies, 45%), with $\dot{V}$O_2max_ (mL/kg/min) being the most prevalent outcome variable (49 studies, 87%). Besides this, Yo-Yo intermittent recovery test level 1 (YYIR1) and level 2 (YYIR2) were the other two most frequently used tests, identified in 22 (18%) and ten studies (8%), respectively. Distance in metres was the most common outcome variable in both tests, reported in 20 studies (91%) in YYIR1 and in ten studies (100%) in YYIR2.

**Table 2:** Maximal aerobic tests and outcome variables

Test	Test type	Outcome variable	Reference
Incremental treadmill Decltest to exhaustion	Laboratory	$\dot{V}$O_2max_ (mL/kg/min)	[15, 18, 22, 34, 36, 56, 59, 62, 67, 83–90, 112, 116, 120, 130, 131, 133, 135, 140, 143, 149, 159, 160, 162–166, 168, 173, 175–177, 179, 183–185, 188, 192, 198–201]
		HR_max_ (b/min)	[18, 34, 56, 59, 62, 67, 84, 89, 112, 135, 149, 159, 166, 175, 179, 192, 200]
		Velocity at $\dot{V}$O_2max _(km/h)	[19, 120, 135, 143, 173, 176, 177, 179, 192]
		Blood lactate concentration (mmol/L)	[18, 62, 88, 112, 135, 149, 166, 192]
		$\dot{V}$O_2max_ (mL/lbm/min)	[190]
		$\dot{V}$O_2max_ (L/min)	[19, 184]
		$\dot{V}$O_2peak_ (mL/kg/min)	[195]
		$\dot{V}$O_2_ at 12 km/h (mL/kg/min)*	[59]
		$\dot{V}$O_2_ at respiratory compensation point (L/min)*	[184]
		$\dot{V}$O_2_ at respiratory compensation point (mL/kg/min)*	[184]
		$\dot{V}$O_2_ at ventilatory threshold (mL/kg/min)*	[36]
		$\dot{V}$O_2_ at lactate threshold (mL/kg/min)*	[36]
		$\dot{V}$O_2_ at anaerobic threshold (mL/kg/min)*	[59, 84, 185]
		$\dot{V}$CO_2max_ (mL/kg/min)	[56]
		Lactate threshold (mmol/L)*	[34, 151]
		Velocity at lactate threshold (km/h)*	[34, 151, 198]
		HR at lactate threshold (b/min)*	[34, 151]
		Ventilatory threshold*	[120, 135]
		Velocity at 2 mmol/L (km/h)*	[61, 62, 130, 165, 197]
		Velocity at 2.5 mmol/L (km/h)*	[61]
		Velocity at 3 mmol/L (km/h)*	[61]
		Velocity at 4 mmol/L (km/h)*	[61, 62, 130, 160, 164, 165, 176, 189, 197]
		Ventilatory threshold 1 (mL/kg/min)*	[143]
		Ventilatory threshold 2 (mL/kg/min)*	[143, 188]
		%$\dot{V}$O_2max_ at ventilatory threshold 1 (%)*	[86, 143]
		%$\dot{V}$O_2max_ at ventilatory threshold 2 (%)*	[86, 143]
		%$\dot{V}$O_2max_ at 4 mmol/L (%)*	[176]
		$\dot{V}$O_2_ at 2 mmol/L (mL/kg/min)*	[62]
		$\dot{V}$O_2_ at 4 mmol/L (mL/kg/min)*	[62]
		%HR_max_ at 4 mmol/L (%)*	[176]
		%$\dot{V}$O_2max_ at ventilatory threshold (%)*	[89, 160, 177]
		%$\dot{V}$O_2max_ at anaerobic threshold (%)*	[190, 200, 201]
		$\dot{V}$O_2_ at ventilatory threshold (mL/kg/min)*	[160, 164]
		$\dot{V}$O_2rest_ (mL/kg/min)	[86]
		Time to exhaustion (min)	[85, 87, 120, 133, 159, 173]
		Maximum speed (km/h)	[18, 56, 84, 159]
		Maximal ventilation (L/min)	[149]
		Running economy (mL/kg/min)*	[34, 133]
		Running economy (mL/lbm/min)*	[190]
		Time to reach a respiratory exchange ratio of 1 (s)*	[133]
		Respiratory compensation point*	[120]
		Maximal respiratory exchange ratio	[149, 175]
		HR at ventilatory threshold (b/min)*	[120]
		HR at 2 mmol/L (b/min)*	[61, 62, 197]
		HR at 2.5 mmol/L (b/min)*	[61]
		HR at 3 mmol/L (b/min)*	[61]
		HR at 4 mmol/L (b/min)*	[61, 62, 197]
		HR at 12 km/h (b/min)*	[59]
		HR at anaerobic threshold (b/min)*	[59, 84]
		HR at respiratory compensation point (b/min)*	[120]
		HR at $\dot{V}$O_2max_ (b/min)	[120]
		Velocity at anaerobic threshold (km/h)*	[140] [56, 84, 88]
		Velocity at ventilatory threshold (km/h)*	[120, 135, 177]
		Velocity at ventilatory threshold 1 (km/h)	[143]
		Peak aerobic speed (km/h)	[116]
		Aerobic threshold (km/h)*	[116]
		Anaerobic threshold (km/h)*	[116]
		Velocity at respiratory compensation point (km/h)*	[120]
Yo-Yo intermittent recovery test level 1	Field	Distance (m)	[15–17, 24–26, 91, 92, 111, 114, 115, 117, 136, 137, 141, 148, 154, 164, 165, 181]
		HR_max_ (b/min)	[15, 110]
		Final stage velocity (km/h)	[136]
		HR at 2nd minute of the test (b/min)*	[25]
		HR at 4th minute of the test (b/min)*	[25]
		$\dot{V}$O_2max_ (mL/kg/min)	[119]
Yo-Yo intermittent recovery test level 2	Field	Distance (m)	[15, 25, 27, 42, 93, 139, 155, 159, 173, 179]
		HR_max_ (b/min)	[15, 159]
		HR at 2nd minute of the test (b/min)*	[25]
		HR at 4th minute of the test (b/min)*	[25]
VAMEVAL test	Field	MAS (km/h)	[144, 152, 157, 169, 181]
		HR_max_ (b/min)	[144, 147, 152, 181]
		$\dot{V}$O_2max_ (mL/kg/min)	[165, 169]
		Blood lactate concentration (mmol/L)	[147]
		Velocity at last stage (km/h)	[147]
		HR_mean_ (b/min)	[169]
		Time to exhaustion (min)	[121]
		Distance (m)	[181]
Yo-Yo intermittent endurance test level 2	Field	Distance (m)	[67, 94, 95, 167, 170, 174, 177, 180]
		HR_max_ (b/min)	[67, 167, 174]
		HR_mean_ (b/min)	[167, 174]
20-m multistage fitness test (beep test)	Field	Distance (m)	[26, 132, 134]
		MAS (km/h)	[134]
		HR_max_ (b/min)	[134, 194, 196]
		Number of runs	[196]
		Resting blood lactate concentration (mmol/L)	[134]
		Post-exercise blood lactate concentration (mmol/L)	[134]
		Time to exhaustion (min)	[134]
		$\dot{V}$O_2max_ (mL/kg/min)	[134, 186, 194]
30–15 Intermittent Fitness Test	Field	Final velocity (km/h)	[13, 68, 121, 124, 138, 146]
		HR_max_ (b/min)	[121, 138]
Modified Yo-Yo intermittent recovery test level 1	Field	Distance (m)	[128]
University of Montreal Track Test	Field	Time to exhaustion (min)	[118]
		Distance (m)	[118]
		MAS (km/h)	[118, 193]
		HR_max_ (b/min)	[193]
		$\dot{V}$O_2max_ (mL/kg/min)	[118]
		Anaerobic speed reserve (km/h)	[118]
Modified University of Montreal Track Test	Field	Final velocity (km/h)	[178, 187]
		MAS (km/h)	[182]
		HR_max_ (b/min)	[178]
		Blood lactate concentration (mmol/L))	[178]
2-phase progressive treadmill test	Laboratory	HR_max_ (b/min)	[113]
		Blood lactate concentration (mmol/L)	[113]
		Velocity at 4 mmol/L (km/h)*	[113]
1000-m time trial	Field	Time to completion (s)	[137]
1500-m time trial	Field	MAS (m/s)	[109]
Progressive maximal field test to exhaustion	Field	MAS (km/h)	[160]
Conconi test	Field	MAS (km/h)	[123]
Intermittent endurance running (INTER) soccer specific test	Field	Time to exhaustion (s)	[166]
		Distance (m)	[166]
		Peak blood lactate concentration (mmol/L)	[166]
		Mean blood lactate concentration (mmol/L)	[166]
		HR_peak_ (b/min)	[166]
		HR_mean_ of the last 15 s (b/min)	[166]
Incremental running test	Field	Velocity at lactate threshold (m/s)	[132]
Incremental endurance test	Laboratory	Velocity at 2 mmol/L (km/h)*	[125]
		Velocity at 4 mmol/L (km/h)*	[125, 168]
		HR_max_ (b/min)	[125]
		Velocity at lactate threshold (km/h)*	[168]
		HR at velocity at 2 mmol/L (%max)*	[125]
		HR at velocity at 4 mmol/L (%max)*	[125]
		Distance (m)	[125]
Interval shuttle run test	Field	Distance (m)	[125, 168]
		HR_max_ (b/min)	[125, 196]
		Number of runs	[196]
Cooper 12 min test	Field	Distance (m)	[200]
Bangsbo test	Field	Distance (m)	[192, 199]

Outcome variables marked with an asterisk (*) represent submaximal variables

*HR* heart rate, *HR*_*max*_ maximum heart rate, *HR*_*mean*_ average heart rate, *HR*_*peak*_ peak heart rate, *MAS* maximal aerobic speed, $\dot{V}$*CO*_*2max*_ maximum carbon dioxide production, $\dot{V}$*O*_*2*_ oxygen uptake, $\dot{V}$O_2*max*_ maximum oxygen uptake, $\dot{V}$*O*_*2peak*_ peak oxygen uptake, *O*_*2rest*_ ventilatory efficiency at rest

**Table 3:** Submaximal aerobic tests and outcome variables

Test	Test type	Outcome variable	Reference
Submaximal Yo-Yo intermittent recovery test level 1	Field	%HR_max_ (%) at 3 min	[110]
		%HR_max_ (%) at 6 min	[110]
		%HR_max_ (%) at 1 min of recovery	[110]
		%HR_max_ (%) at 2 min of recovery	[110]
		HR_mean_ during the last 30 s (%HR_max_)	[129]
		HR at 6 min (completion of test) (b/min)	[66]
		HR at 30 s after completion (b/min)	[66]
		HR at 60 s after completion (b/min)	[66]
		HR at 90 s after completion (b/min)	[66]
		HR at 120 s after completion (b/min)	[66]
		HRR at 30 s after completion (%HR_max_)	[66]
		HRR at 60 s after completion (%HR_max_)	[66]
		HRR at 90 s after completion (%HR_max_)	[66]
		HRR at 120 s after completion (%HR_max_)	[66]
Submaximal Yo-Yo intermittent endurance test level 2	Field	HRR at 4 min after completion (%HR_max_)	[67]
		HRR at 6 min after completion (%HR_max_)	[67]
Modified lactate threshold treadmill test	Laboratory	Velocity at 2 mmol/L (km/h)	[172]
		HR at 2 mmol/L (km/h)	[172]
		Velocity at 4 mmol/L (km/h)	[172]
		HR at 4 mmol/L (km/h)	[172]
Submaximal lactate threshold treadmill test	Laboratory	Velocity at first lactate inflection point (km/h)	[191]
		Velocity at 4 mmol/L (km/h)	[191]
Submaximal treadmill test	Laboratory	$\dot{V}$O_2_ (mL/kg/min)	[171]
		Respiratory exchange ratio	[171]
		Respiratory rate	[171]
		HR at 9, 11, 14 km/h (b/min)	[171]
		Blood lactate concentration (mmol/L)	[171]
Submaximal warm-up test	Field	HR exercise (b/min)	[68, 126]
		HR recovery 60 s (b/min)	[68, 126]
		HR post 1 min (b/min)	[68, 126]
4-stage submaximal intermittent running test (around the field)	Field	Velocity at 3 mmol/L (km/h)	[142, 145, 150, 156]
		Blood lactate concentration at 12 km/h (mmol/L)	[150, 156]
		Blood lactate concentration at 13 km/h (mmol/L)	[150, 156]
		Individual anaerobic threshold (km/h)	[161]
Square-wave treadmill protocol	Laboratory	$\dot{V}$O_2_ kinetics	[173]
Mognoni test	Field	Blood lactate concentration (mmol/L)	[123]
Change of direction economy test	Field	$\dot{V}$O_2_ (L/min)	[83]
		Energetic cost (kcal/kg/km)	[83]
		Respiratory exchange ratio	[83]
		$\dot{V}$CO_2_ (L/min)	[83]
		Minute ventilation (breaths/min)	[83]
		Mean HR of the last minute (b/min)	[83]
		Blood lactate concentration (mmol/L)	[83]
		Distance (m)	[125]
4 × 6 submaximal running at 75% MAS	Laboratory	$\dot{V}$O_2_ kinetics	[179]
		Running economy (mL/kg/km)	[179]
		Respiratory exchange ratio	[179]
		HR during the final 30 s of each running bout (%HR_max_)	[179]
Constant speed running test	Field	Blood lactate concentration (mmol/L)	[122]
High-intensity intermittent test	Field	Blood lactate concentration (mmol/L)	[15, 22, 122]
10-min run on treadmill (60% of peak speed)	Laboratory	$\dot{V}$O_2_ kinetics	[15, 22]

*HR* heart rate, *HR*_*max*_ maximum heart rate, *HR*_*mean*_ average heart rate, *HRR* heart rate recovery, *MAS* maximal aerobic speed, $\dot{V}$*CO*_*2*_ carbon dioxide production, $\dot{V}$*O*_*2*_ oxygen uptake, $\dot{V}$*O*_*2*_* kinetics* the rate which $\dot{V}$O_2_ adjusts to a dynamic exercise challenge

To enhance clarity and structure, the identified tests were categorized into maximal and submaximal assessments. A total of 21 maximal tests were identified, 18 of which were field based (Table 2). These were further categorized into continuous protocols (ten tests) and intermittent protocols (eight tests). The identified maximal continuous field tests were the VAMEVAL test, the 20-m multistage fitness test (beep test), the University of Montreal Track Test (UMTT) and its modified version, the 1000-m and the 1500-m time trials, a progressive maximal field test to exhaustion, the Conconi test, an incremental running test, and the Cooper test. The identified maximal intermittent field tests were the YYIR1 and YYIR2 tests, the Yo-Yo intermittent endurance test level 2 (YYIE2), the 30–15 Intermittent Fitness Test (30–15 IFT), a modified version of the YYIR1, an intermittent endurance running soccer specific test, an interval shuttle run test, and the Bangsbo test. Notably, five of the maximal tests, including the incremental treadmill test to exhaustion, the YYIR1 and YYIR2, the 2-phase progressive treadmill test, and the incremental endurance test, also assessed submaximal outcome variables. These variables included threshold parameters (e.g. lactate threshold, anaerobic threshold, ventilatory threshold), performance at specific intensities (e.g. $\dot{V}$O_2_ and HR at certain velocities), and running economy, providing additional insights into aerobic performance.

In addition to the maximal tests, 14 submaximal tests were identified, comprising eight field-based tests and six laboratory-based tests (Table 3). The field-based submaximal test included the 6-min submaximal YYIR1, the submaximal YYIE2, the 4-min submaximal warm-up test, the 4-stage submaximal intermittent running test, the Mognoni test, the change of direction (COD) economy test, the constant speed running test, and the high-intensity intermittent test. The laboratory-based submaximal tests included the modified lactate threshold treadmill test, the submaximal lactate threshold treadmill test, the submaximal treadmill test, the square-wave treadmill protocol, the 4 × 6 submaximal running test at 75% maximal aerobic speed (MAS), and the 10-min run on treadmill (60% of peak speed).

### Tests and Outcome Variables used to Assess Repeated Sprint Ability (RSA)

RSA was assessed in 27 studies (20.6%) (Table 4). A total of 18 different tests were used to assess RSA ability with 17 (95%) of those being running-based, whereas one was performed on a cycle ergometer. In terms of running-based tests, 11 tests (65%) included linear sprint tests, while six (35%) included at least one COD ranging from 45 to 180°. Sprint repetitions ranged from 6 to 15, sprint distances from 20 to 40 m, and duration of recovery period from 10 to 30 s. Overall, active recovery was the most prevalent recovery type (14 tests, 78%), whereas the remaining four (22%) incorporated passive recovery. The 6 × 40-m (20 + 20 m, 180° COD) shuttle sprint protocol with 20 s passive recovery was the most commonly used RSA test (eight studies, 29%), with RSA_best_ in seconds (eight studies, 100%), RSA_mean_ in seconds (eight studies, 100%), and percentage of performance decrement (eight studies, 100%), representing the most frequently examined outcome variables. The 6 × 20-m sprint protocol with 25 s active recovery (used in three studies, 11%), evaluating RSA_best_, RSA_mean_, RSA_total_, and percentage of performance decrement, and a 7 × 30-m sprint protocol with 25 s active recovery (three studies, 11%), assessing RSA_best_, RSA_mean_, fatigue index in seconds, RSA_total_, and percentage of performance decrement, were the other two most frequently used RSA tests.

**Table 4:** RSA tests and outcome variables

Test	Outcome variable	Reference
6 × 40-m (20 + 20) shuttle sprints with 20 s passive recovery	RSA_best_ (s)	[21, 22, 26, 83, 136, 153, 158, 187]
	RSA_mean_ (s)	[21, 22, 26, 83, 136, 153, 158, 187]
	RSA_total_ (s)	[158]
	Performance decrement (%)	[21, 22, 26, 83, 136, 153, 158, 187]
	Blood lactate concentration (mmol/L)	[83, 136]
6 × 20-m sprints with 25 s active recovery	RSA_best_ (s)	[23, 75, 171]
	RSA_mean_ (s)	[23, 75]
	RSA_total_ (s)	[23, 75, 171]
	Performance decrement (%)	[23, 75, 171]
7 × 30-m sprints with 25 s active recovery	RSA_best_ (s)	[139, 173]
	RSA_mean_ (s)	[139, 173]
	Fatigue index (s)	[173]
	RSA_total_ (s)	[24]
	Performance decrement (%)	[24, 139]
6 × 20-m sprints (with 4 changes of directions [100°] every 4 m) with 25 s active recovery	RSA_best_ (s)	[23, 75]
	RSA_mean_ (s)	[23, 75]
	RSA_total_ (s)	[23, 75]
	Performance decrement (%)	[23, 75]
	RSA/RCOD index	[23, 75]
8 × 30-m sprints with 25 s active recovery	RSA_best_ (s)	[92]
	RSA_mean_ (s)	[92]
	RSA_total_ (s)	[92]
	Performance decrement (%)	[92]
Bangsbo sprint test (7 × 34.2-m sprint with 3 changes of directions [45°] with 25 s active recovery)	RSA_best_ (s)	[127]
	RSA_mean_ (s)	[127]
	Fatigue index (s)	[127]
	Performance decrement (%)	[127]
15 × 40-m sprints with 30 s passive recovery	RSA_total_ (s)	[155]
	Performance decrement (%)	[155]
7 × 30-m sprints (completed on 20-s cycles)	RSA_total_ (s)	[27]
	Performance decrement (%)	[27]
10 × 20-m sprints with 15 s active recovery	RSA_best_ (s)	[179]
	RSA_total_ (s)	[179]
	Sprint fatigue index (%)	[179]
10 × 20-m sprints with 25 s active recovery	RSA_best_ (s)	
	RSA_mean_ (s)	[74]
	RSA_total_ (s)	[74]
	Performance decrement (%)	[74]
10 × 20-m sprints (with 4 changes of directions [100°] every 4 m) with 25 s active recovery	RSA_best_ (s)	[74]
	RSA_mean_ (s)	[74]
	RSA_total_ (s)	[74]
	Performance decrement (%)	[74]
	RSA/RCOD index	[74]
6 × 35-m sprints with 10 s active recovery	RSA_best_ (s)	[157]
	RSA_total_ (s)	[157]
	Performance decrement (%)	[157]
	Maximal sprinting speed (km/h)	[157]
7 × 35-m sprints with 25 s active recovery	RSA_mean_ 10 m (s)	[25]
	RSA_mean_ 20 m (s)	[25]
	RSA_mean_ (s)	[25]
6 × 40-m sprints (involving 3 changes of directions) with 25 s passive recovery	RSA_best_ (s)	[170]
	RSA^total^ (s)	[170]
	Performance decrement (%)	[170]
10 × 6-s cycle sprints with 24 s of passive recovery (60% of optimal resistive load)	Peak power (W)	[177]
	Relative peak power (W/lean leg volume)	[177]
	Mean power (W)	[177]
	Relative mean power (W/lean leg volume)	[177]
	Total work (kJ)	[177]
	Performance decrement (%)	[177]
8 × 20-m sprints with 20 s active recovery	RSA_best_ (s)	[186]
	RSA_total_ (s)	[186]
6 × 30-m shuttle sprints with 20 s passive recovery	RSA_mean_ (s)	[131]
6 × 30-m sprints with 30 s active recovery	RSA_best_ (s)	[140]
	RSA_total_ (s)	[140]
	Performance decrement (%)	[140]

*RCOD* repeated change of direction, *RSA* repeated sprint ability, *RSA*_*best*_ best sprint time, *RSA*_*mean*_ mean sprint time, *RSA*_*total*_ total sprint time

### Normative Values for Aerobic Fitness in Elite Male Soccer Players

Table 5 presents the normative values for $\dot{V}$O_2max_ obtained in incremental treadmill testing to exhaustion. $\dot{V}$O_2max_ values ranged from 51.5 to 65.5 mL/kg/min in senior professionals (37 studies, 84%), while the observed range in elite youth was 54.9 to 63.5 mL/kg/min (seven studies, 16%). Table 6 provides the normative values for YYIR1 and YYIR2. In terms of YYIR1, senior professionals achieved distances ranging from 1348 to 2520 m (14 studies, 78%), whereas distances in elite youth ranged from 2054 to 3150 m (six studies, 33%). For YYIR2, senior professionals reached distances ranging from 613 to 1300 m (seven studies, 70%), whilst distances in elite youth ranged from 815 to 1147 m (five studies, 50%). Table 7 provides normative values for other frequently used field tests in elite male soccer players. For the VAMEVAL test, MAS values in senior professionals ranged from 15.9 to 19.6 km/h (five studies, 62%). Regarding the YYIE2, senior professionals achieved distances ranging from 1640 to 2364 m (five studies, 62%), while distances in elite youth were between 1394 and 2892 m (two studies, 25%). In the 20-m multistage fitness test (beep test), senior professionals achieved distances ranging from 1981 to 2595 m (three studies, 50%). Lastly, the final velocity in 30–15 IFT ranged from 19.0 to 20.1 km/h in senior professionals (two studies, 33%), whereas the single study identified in elite youth players reported a final velocity of 19.5 km/h.

**Table 5:** Normative values for $\dot{V}$O_2max_ (mL/kg/min) during incremental treadmill tests to exhaustion

Study	Playing standard	Playing position/subgroup	Protocol	$\dot{V}$O_2max_ (mL/kg/min)
Boraczyński et al. [112]	Senior professionals	All	Continuous 3-min running stages (at 1% gradient) with initial speed set at 9 km/h increased by 1 km/h per stage until volitional exhaustion (failure) was reached	55.4 ± 5.3
Dolci et al. [83]	Elite youth	All	Running started at 10 km/h, and speed was increased by 2 km/h every 3 min until volition	63.5 ± 7.7
Parpa and Michaelides [85]	Senior professionals	All	Modified Heck incremental maximal protocol	57.7 ± 1.4
Enes et al. [116]	Senior professionals	All	The test started with 8 km/h with a progressive increase of 0.1 km/h every 6 s, with a steady slope of 1%	54.5 ± 3.8
Angoorani et al. [86]	Senior professionals	All	Bruce protocol → Running started at 1.7 mph (10% grade). The speed of running was subsequently increased to 2.5 mph, 3.4 mph, 4.2 mph, 5.0 mph, and 5.5 mph at 3-min intervals throughout the test. The inclination of the treadmill was also enhanced by 2% for each stage of test	55.3 ± 5.4
Colosio et al. [62]	Senior professionals	All (excluding goalkeepers)	Two-part step-incremental running test → The first, submaximal part of the protocol consisted of steps of 3 min starting from a speed of 8 km/h, which increased with 2 km/h after each step. When blood lactate concentration had passed the threshold of 4 mmol/L, the second, maximal part of the protocol started, and speed was increased by 1 km/h, while the slope of the treadmill was increased 0.5% every 30 s until volitional exhaustion	58.3 ± 3.8
Papadakis et al. [130]	Senior professionals	All	The initial speed of the incremental test was set at 10 km/h and was increased by 2 km/h every 3 min until volitional exhaustion	59.7 ± 3.7
Hoppe et al. [133]	Elite youth	U21 group	After a 4-min run at 10 km/h with 1% inclination, the latter was increased to 5% for further 4 min. Then, the speed was increased by 1 km/h every 2 min until maximal exhaustion was reached	55.0 ± 1.7
		U19 group		54.9 ± 1.3
Krespi et al. [131]	Elite youth	All	The starting speed was 3 km/h, with speed increments of 1 km/h every 60 s Participants walked the first 5 steps (up to 7 km/h) and continued running from 8 km/h until volitional exhaustion	56.2 ± 4.7
Bekris et al. [135]	Senior professionals	All		56.1
Meckel et al. [140]	Senior professionals	All	Treadmill’s initial speed was 9 km/h with a 1% grade. The speed was increased by 1 km/h every minute until volitional exhaustion occurred. The grade was maintained at 1% throughout the entire test until volitional exhaustion	57.4 ± 5.4
Almeida et al. [143]	Senior professionals	Control group	Modified Heck incremental maximal protocol	56.9 ± 4.2
Michaelides et al. [87]	Senior professionals	First Division group	Modified Heck incremental maximal protocol	57.0 ± 5.5
		Second Division group		52.2 ± 5.4
Sapp et al. [149]	Elite youth	All	All players ran at a speed of 12.9 km/h or 13.7 km/h. The players ran at a 0.5% incline for the first 2 min of the test, after which incline increased 2% every subsequent 2 min until volitional exhaustion	56.9 ± 5.1
Wells et al. [159]	Senior professionals	All		57.3 ± 4.5
Manzi et al. [160]	Senior professionals	All	4–5 submaximal exercise bouts at an initial running speed of 9 km/h followed by a maximal incremental test to volitional fatigue. The treadmill running velocity was increased during the submaximal test by 1 km/h every 5 min. Once capillary blood lactate concentrations were elevated above 4 mmol/L, the treadmill speed was increased by 0.5 km/h every 30 s until exhaustion	59.2 ± 4.3
Koundourakis et al. [162]	Senior professionals	All	Initial speed was set at 10 km/h, and it was held constant for 3 min. Thereafter, speed was increased by 2 km/h every 3 min until 16 km/h, and then speed was increased 2 km/h every 2 min until volitional exhaustion	60.1 ± 3.3
Koundourakis et al. [163]	Senior professionals	All	Initial speed was set at 10 km/h, and it was held constant for 3 min. Thereafter, speed was increased by 2 km/h every 3 min until 16 km/h, and then speed was increased 2 km/h every 2 min until volitional exhaustion	58.0 ± 3.2
Manzi et al. [164]	Senior professionals	All	Starting speed of 10 km/h and speed increments of 1 km/h/min until exhaustion	61.2 ± 4.1
Castagna et al. [165]	Senior professionals	All	4–5 submaximal exercise bouts at an initial running speed of 9 km/h followed by a maximal incremental test to volitional fatigue. The treadmill running velocity was increased during the submaximal test by 1 km/h every 5 min. Once capillary blood lactate concentrations were elevated above 4 mmol/L, the treadmill speed was increased by 0.5 km/h every 30 s until exhaustion	61.4 ± 4.1
Aandstad and Simon [166]	Senior professionals	All	Running started at 12 km/h (5.2% inclination). Treadmill speed was increased by 1 km/h every minute until volitional exhaustion	59.9 ± 2.1
Hoppe et al. [168]	Senior professionals	All	After a 4-min run at 10 km/h with 1% inclination, the inclination was increased to 5% for 4 min. The treadmill speed was than increased every 2 min by increments of 1 km/h until exhaustion was reached	58.2 ± 4.9
Boone et al. [88]	Senior professionals	All (excluding goalkeepers)	The incremental exercise consisted of steps of 3 min starting from a speed of 8 km/h, and the speed was increased with 2 km/h after each step (1.5% slope)	58.5 ± 3.0
Signorelli et al. [59]	Senior professionals	All	After 1 min at 5.5 km/h, speed was increased to 8 km/h and thereafter increased by 0.1 km/h every 7.5 s (0.8 km/h per min) until exhaustion	63.3 ± 6.2
Angius et al. [56]	Senior professionals	All	Linear increase in running velocity of 1 km/h every minute, starting at a speed of 8 km/h up to exhaustion	55.2 ± 4.6
Wells et al. [173]	Senior professionals	All (professional group)		56.5 ± 2.9
Helgerud et al. [175]	Senior professionals	All	Players started at 11 km/h and kept there for 5 min. The speed of the treadmill was then increased by 1 km/h every minute to a level that brought the participant to exhaustion within 5–6 min	60.5 (51.7–67.1)
Kalapotharakos et al. [176]	Senior professionals	All	Initial speed was set at 10 km/h, and it was held constant for 3 min. Thereafter, speed was increased by 2 km/h every 3 min until 16 km/h, and then speed was increased 2 km/h every 2 min until volitional exhaustion	61.2 ± 2.3
Bogdanis et al. [177]	Senior professionals	All	Speed was increased by 0.5 km/h every minute, starting from a speed of 10–12 km/h	51.5 ± 1.7
Ziogas et al. [34]	Senior professionals	First division group	The initial speed of the incremental test was set at 10 km/h and was increased by 2 km/h/min every 3 min until volitional exhaustion	58.8 ± 3.3
		Second division group		56.4 ± 3.7
		Third division group		57.6 ± 3.2
Rampinini et al. [15]	Senior professionals	All	Starting speed at 10 km/h, with increments of 1 km/h/min until volitional exhaustion (4% inclination)	58.5 ± 3.8
Rampinini et al. [22]	Senior professionals	All	Starting speed at 10 km/h, with increments of 1 km/h/min until volitional exhaustion (4% inclination)	58.5 ± 4.0
Sotiropoulos et al. [183]	Senior professionals	All	The speed started at 10 km/h, and was increased by 1 km/h every 2 min until volitional exhaustion	57.8 ± 2.6
Sporis et al. [18]	Senior professionals	All	1-min incremental maximal exercise test	60.1 ± 2.3
Voutselas et al. [188]	Senior professionals	All	Protocol started with 15-min warm-up at 50% of their $\dot{V}$O_2max_ (of previously estimated $\dot{V}$O_2max_ tests) followed by a 3-min rest, after which the athletes started to run at approximately 60% of their $\dot{V}$O_2max_. Intensity was increased by 0.5 km/h every 1 min until volitional exhaustion	52.3 ± 5.0
Chamari et al. [192]	Elite youth	All	Participants ran for 3 min at 9 km/h. The speed was then increased by 1 km/h every minute until exhaustion, which occurred within 10–15 min	61.1 ± 4.6
Edwards et al. [36]	Senior professionals	All	A series of incremental steps, which increased in speed every 3.5 min to a maximum of 4.03 m/s. After the final 3 min stage at 4.03 m/s was completed, the incline of the treadmill was increased by 2% every minute	62.1 ± 4.9
Helgerud et al. [198]	Elite youth	All	Treadmill speed started at 11 km/h and kept there for 5 min. The speed of the treadmill was then increased by 1 km/h every minute to a level that brought the participant to exhaustion within 5–6 min (constant inclination at 5.5%)	58.2 ± 4.4
Casajús [84]	Senior professionals	All	The test began at a 3% grade and a speed of 8 km/h. The grade was held constant, and the speed increased 1 every min until exhaustion	65.5 ± 8.0
Al-Hazzaa et al. [89]	Senior professionals	All	Following a 6-min warm-up period, the athlete began running while the treadmill speed was gradually increased until a velocity of 15.5 km/h was reached, after which the treadmill velocity was kept constant and the inclination was increased by 2% every 2 min until volitional exhaustion	56.8 ± 4.8
Wisløff et al. [90]	Senior professionals	All	The speed of the treadmill was increased every minute to a level that brought the participant close to exhaustion after approximately 5 min. Inclination was constant at 3°. Immediately after $\dot{V}$O_2max_ determination, each participant ran for 2 min at an exercise intensity of 50–60% of $\dot{V}$O_2max_ directly followed by a supramaximal intensity run, resulting in exhaustion after ≈ 3 min	63.8 ± 4.1
Bangsbo and Lindquist [199]	Senior professionals	All	The initial treadmill speed was 18 km/h, and it was increased 2 km/h every 2 min until the participant was exhausted	60.8 ± 1.3
Chin et al. [200]	Senior professionals	All	After a 10-min warm-up at 8.0 km/h (0% grade), the participant began running at a velocity of 12.1 km/h (0% grade). Every 2 min thereafter, the grade was increased by 2.5% until volitional exhaustion	59.1 ± 4.9
Rhodes et al. [201]	Elite youth	All	After a 10-min warm-up at 8.0 km/h (0% grade), the participant began running at a velocity of 12.1 km/h (0% grade). Each minute thereafter the treadmill velocity was increased by 0.8 km/h until volitional exhaustion	58.7 ± 4.1

Data are presented as mean ± standard deviation

$\dot{V}$*O*_*2max*_ maximum oxygen uptake

**Table 6:** Normative values for distance (m) during YYIR1 and YYIR2

Study	Test	Playing standard	Playing position/subgroup	Distance (m)
Akyildiz et al. [114]	YYIR1	Senior professionals	All	2083 ± 404
Schons et al. [115]	YYIR1	Senior professionals	All (men)	2271 ± 744
Arregui-Martin et al. [117]	YYIR1	Elite youth	All	2716 ± 242
Owen et al. [91]	YYIR1	Senior professionals	All	1349 ± 167
Saidi et al. [136]	YYIR1	Senior professionals	All	2520 ± 363
Clancy et al. [137]	YYIR1	Elite youth	All	2138 ± 293
Rago et al. [141]	YYIR1	Senior professionals	All	2260 ± 277
Rodríguez-Fernández et al. [92]	YYIR1	Senior professionals	Senior professionals group	2368 ± 265
		Elite youth	Elite youth group	2054 ± 289
Pareja-Blanco et al. [148]	YYIR1	Senior professionals	All	1500 ± 419
Pareja-Blanco et al. [26]	YYIR1	Senior professionals	All	1558 ± 362
Noon et al. [154]	YYIR1	Elite youth	All	3150 ± 269
Ingebrigtsen et al. [25]	YYIR1	Senior professionals	All	1736 ± 443
Manzi et al. [164]	YYIR1	Senior professionals	All	2366 ± 409
Castagna et al. [165]	YYIR1	Senior professionals	All	2390 ± 409
Chaouachi et al. [24]	YYIR1	Elite youth	All	2289 ± 409
Wong et al. [181]	YYIR1	Senior professionals	All	1525 ± 63
Rampinini et al. [15]	YYIR1	Senior professionals	All	2231 ± 294
Mujika et al. [16]	YYIR1	Senior professionals	All	2414 ± 456
		Elite youth	All	2092 ± 260
Lockie et al. [27]	YYIR2	Elite youth	All	1062 ± 371
Enright et al. [139]	YYIR2	Elite youth	All	920 ± 156
Stevens et al. [93]	YYIR2	Senior professionals	Senior professionals group	1300 ± 210
		Elite youth	Elite youth group	1147 ± 244
Iaia et al. [155]	YYIR2	Elite youth	All	958 ± 208
Wells et al. [159]	YYIR2	Senior professionals	All	893 ± 42
Ingebrigtsen et al. [25]	YYIR2	Senior professionals	All	613 ± 174
Wells et al. [173]	YYIR2	Senior professionals	All	966 ± 153
Christensen et al. [179]	YYIR2	Senior professionals	All	891 ± 131
Rampinini et al. [15]	YYIR2	Senior professionals	All	958 ± 99
Krustrup et al. [42]	YYIR2	Senior professionals	International elite group	1050
		Elite youth	Elite U19	815

Data are presented as mean ± standard deviation

*YYIR1* Yo-Yo intermittent recovery test level 1, *YYIR2* Yo-Yo intermittent recovery test level 2

**Table 7:** Normative values for MAS (km/h) during VAMEVAL, distance (m) during YYIE2, distance (m) during 20-m multistage fitness test (beep test), and final velocity (km/h) during 30–15 IFT

Study	Test	Playing standard	Playing position/subgroup	MAS (km/h)	Distance (m)	Final velocity (km/h)
Fessi et al. [144]	VAMEVAL	Senior professionals	All	17.6 ± 2.3		
Fessi et al. [152]	VAMEVAL	Senior professionals	All	17.8 ± 0.9		
Brocherie et al. [157]	VAMEVAL	Senior professionals	All	17.1 ± 1.3		
Lago-Ballesteros [169]	VAMEVAL	Senior professionals	All	19.6 ± 0.8		
Wong et al. [181]	VAMEVAL	Senior professionals	All	15.9 ± 0.2		
Bradley et al. [94]	YYIE2	Senior professionals	Premier League group		2364 ± 478	
			Championship group		2268 ± 567	
			League 1 group		2226 ± 432	
Rebelo et al. [95]	YYIE2	Elite youth	All		1394 ± 421	
Silva et al. [167]	YYIE2	Senior professionals	All		1776 ± 358	
Gibson et al. [170]	YYIE2	Elite youth	U19 group		2892 ± 484	
Silva et al. [174]	YYIE2	Senior professionals	All		1640 ± 196	
Bogdanis et al. [177]	YYIE2	Senior professionals	All		1658 ± 156	
Henderson et al. [180]	YYIE2	Senior professionals	All		2183 ± 401	
Radzimiński et al. [132]	20-m multistage fitness test (beep test)	Senior professionals	All		2561 ± 264	
Boraczyński et al. [134]	20-m multistage fitness test (beep test)	Senior professionals	All		2595 ± 257	
Pareja-Blanco et al. [26]	20-m multistage fitness test (beep test)	Senior professionals	All		1981 ± 309	
Rabbani et al. [124]	30–15 IFT	Senior professionals	First team group			19.0 ± 1.0
		Elite youth	U19 group			19.5 ± 0.7
Campos-Vazquez et al. [146]	30–15 IFT	Senior professionals	All			20.1 ± 0.8

Data are presented as mean ± standard deviation

*30–15 IFT* 30–15 intermittent fitness test, *MAS* maximal aerobic speed, *YYIE2* Yo-Yo intermittent endurance test level 2

### Normative Values for RSA in Elite Male Soccer Players

Table 8 shows the normative values for the most common RSA tests. For the 6 × 40-m (20 + 20 m) shuttle sprint protocol with 20 s passive recovery test, senior professionals achieved values ranging from 6.86 to 7.40 s for RSA_best_ (five studies, 62%), 7.12 to 8.07 s for RSA_mean_ (five studies, 62%), and 3.3% to 7.8% for performance decrement (five studies, 62%). Values in elite youth ranged from 7.02 to 7.38 s for RSA_best_ (three studies, 37%), 7.43 to 7.67 s for RSA_mean_ (three studies, 37%), and 3.7% to 6.6% for performance decrement (five studies, 62%). For 6 × 20-m sprints with 25 s active recovery test, values in senior professionals ranged from 2.89 to 3.14 s for RSA_best_ (three studies, 100%), 17.79 to 19.31 s for RSA_total_ (three studies, 100%), and 1.75% to 2.59% for performance decrement (three studies, 100%).

**Table 8:** Normative values for RSA_best_ (s), RSA_mean_ (s), RSA_total_ (s), and performance decrement (%) for common RSA tests

Study	Test	Playing standard	Playing position/subgroup	RSA_best_ (s)	RSA_mean_ (s)	RSA_total_ (s)	Performance decrement (%)
Dolci et al. [83]	6 × 40-m (20 + 20) shuttle sprints with 20 s passive recovery	Elite youth	All	7.13 ± 0.29	7.43 ± 0.23		3.7 ± 0.01
Saidi et al. [136]	6 × 40-m (20 + 20) shuttle sprints with 20 s passive recovery	Senior professionals	All	7.40 ± 0.40	8.07 ± 0.30		7.8 ± 4.7
Spineti et al. [153]	6 × 40-m (20 + 20) shuttle sprints with 20 s passive recovery	Elite youth	All	7.02 ± 0.17	7.48 ± 0.16		6.6 ± 2.0
Pareja-Blanco et al. [26]	6 × 40-m (20 + 20) shuttle sprints with 20 s passive recovery	Senior professionals	All	7.36 ± 0.18	7.60 ± 0.17		3.3 ± 1.5
Haddad et al. [158]	6 × 40-m (20 + 20) shuttle sprints with 20 s passive recovery	Elite youth	All	7.38 ± 0.14	7.67 ± 0.15	46.01 ± 0.91	3.9 ± 1.6
Rampinini et al. [22]	6 × 40-m (20 + 20) shuttle sprints with 20 s passive recovery	Senior professionals	All	6.86 ± 0.13	7.17 ± 0.09		4.5 ± 1.9
Impellizzeri et al. [21]	6 × 40-m (20 + 20) shuttle sprints with 20 s passive recovery	Senior professionals	All	6.88 ± 0.19	7.12 ± 0.17		3.3 ± 1.5
Rampinini et al. [187]	6 × 40-m (20 + 20) shuttle sprints with 20 s passive recovery	Senior professionals	All	7.00 ± 0.19	7.25 ± 0.17		3.3 ± 1.6
Wong et al. [75]	6 × 20-m sprints with 25 s active recovery	Senior professionals	All	2.89 ± 0.09	2.97 ± 0.11	17.79 ± 0.64	1.75 ± 0.98
		Elite youth	U19 group	2.93 ± 0.09	2.99 ± 0.10	17.91 ± 0.58	2.51 ± 1.17
Owen et al. [171]	6 × 20-m sprints with 25 s active recovery	Senior professionals	All	3.08 ± 0.11		18.96 ± 0.68	2.43 ± 1.42
Wong et al. [23]	6 × 20-m sprints with 25 s active recovery	Senior professionals	All	3.14 ± 0.09	3.22 ± 0.08	19.31 ± 0.47	2.59 ± 1.02
Enright et al. [139]	7 × 30-m sprints with 25 s active recovery	Elite youth	All	4.33 ± 0.19	4.58 ± 0.15		10.6 ± 4.3
Chaouachi et al. [24]	7 × 30-m sprints with 25 s active recovery	Elite youth	All			31.21 ± 1.13	4.0 ± 1.1

Data are presented as mean ± standard deviation

*RSA* repeated sprint ability, *RSA*_*best*_ best sprint time, *RSA*_*mean*_ mean sprint time, *RSA*_*total*_ total sprint time

### Reliability Data

Reliability statistics for the aerobic fitness and RSA tests are shown in Supplementary Tables S4 and S5 of the ESM. For the aerobic fitness tests, reliability statistics were reported in 11 studies (9.6%). The CV (eight studies, 72%), ICC (nine studies, 83%), SEM (three studies, 25%), and MDC (three studies, 25%) were the identified reliability metrics for aerobic fitness testing. YYIR2 was the test in which the greatest number of studies reported reliability values (three studies, 25%). For the total distance achieved in the YYIR2 test, the observed CV values ranged from 4.2% to 9.6%, with an ICC of 0.96, SEM of 34 m, and MDC 94.1 m. Although no study reported reliability values for the distance covered in the YYIR1, excellent reliability was reported for HR at the second (ICC 0.92, CV 4.1%) and fourth (ICC 0.93, CV 3.8%) minutes of the protocol. High levels of reliability were also reported for the 1000-m time trial (ICC 0.82, CV 1.06%, SEM 2.86 s, MDC 4.56 s). The HR_ex_ appeared to be more reliable than HR recovery in the 4-min submaximal warm-up test (ICC 0.95, CV 1.4% vs. ICC 0.84, CV 7.0%) and the 6-min submaximal version of YYIR1 (ICC 0.96, CV 1.6% vs. ICC 0.58–0.93, CV 3.9–19.5%).

For RSA tests, reliability values were reported in five studies (18%). The CV (five studies, 100%), ICC (four studies, 80%), and SEM (one study, 25%) were the reliability metrics reported for RSA tests. Both RSA_total_ (ICC 0.92, CV 0.7–2.7%) and RSA_mean_ (ICC 0.81–0.93, CV 0.8–1.8%) displayed good reliability. Conversely, the performance decrement score showed poor reliability levels (ICC 0.17, CV 30.2%, SEM 1.2%), rendering its utility in practice questionable.

## Discussion

The aims of this systematic review were to (1) identify the tests and outcome variables used to assess aerobic fitness and RSA in elite male soccer players, (2) report normative values on the most common aerobic fitness and RSA tests, and (3) report reliability data for the identified tests and outcome variables. In total, 131 studies from 32 different countries were included in this review. A considerably larger number of studies (i.e. 124 vs. 27) assessed aerobic fitness compared to RSA, which is in agreement with the findings of a recent survey on fitness testing practices of elite male soccer practitioners [45]. This prevalence may be attributed to the fact that RSA research emerged later, with a 20-year gap between the first studies on aerobic fitness and RSA in our review. For aerobic fitness testing, 35 different testing protocols were identified, with the majority of them being field-based tests. However, the incremental treadmill test to exhaustion was the most used aerobic fitness test, followed by the YYIR1 and YYIR2. For RSA testing, 18 different tests were identified, comprising repeated linear sprints and involving at least one COD test, active and passive recovery, different sprint repetitions and distances, as well as recovery durations. The 6 × 40-m (20 + 20 m, 180° COD) shuttle sprint protocol with 20 s passive recovery test was the most commonly used RSA test, followed by a 6 × 20-m sprint protocol with 25 s active recovery and a 7 × 30-m sprint protocol with 25 s active recovery.

### Testing Methods and Outcome Variables

The significant aerobic demands and the need to repeatedly perform short sprints during a 90-min game [6, 7, 39] require the presence of increased aerobic capacity and RSA levels to support a consistent level of performance throughout the game [52]. A valid, reliable, and standardized evaluation of these physical attributes can allow for an objective assessment at both the individual and group level, enabling meaningful within- and between-athlete comparisons to be made [53, 54]. Ultimately, information gained from these assessments can form the basis for the prescription and implementation of tailored training interventions [32].

#### Aerobic Fitness Assessment

Incremental treadmill testing to exhaustion represents the most frequent testing method for aerobic assessment according to the results of this systematic review, with $\dot{V}$O_2max_ being the main outcome variable of interest. A wide range of incremental treadmill testing protocols were used across the studies, varying in terms of initial speed, speed increments, and inclination levels, highlighting the lack of a universal standardized protocol [55]. The role of $\dot{V}$O_2max_ in differentiating between playing standards is questionable, with research in elite soccer yielding equivocal results [15, 19, 34, 56], which may necessitate obtaining additional information from supplementary $\dot{V}$O_2max_ variables [57, 58]. Based on this systematic review, maximum HR (b/min), velocity at $\dot{V}$O_2max_ (km/h), and blood lactate concentration (mmol/L) represent the three most frequently used supplementary outcome variables to gain a more holistic view of an athlete’s aerobic ability. Additionally, outcome variables related to running economy, such as $\dot{V}$O_2_, velocity, and HR at the anaerobic [59, 60] and lactate thresholds [34, 36], as well as at specific intensities [61, 62], were also identified. Velocity at $\dot{V}$O_2max_ integrates aerobic capacity and aerobic cost of running, providing a practical measure that can be used for exercise prescription [63]. Rebelo et al. [58] reported that only velocity at $\dot{V}$_2max_ from incremental treadmill testing correlated with match high-intensity running in youth soccer players, whereas $\dot{V}$O_2max_ showed no significant relationships. Blood lactate concentration provides insights into an athlete’s aerobic ability, with the lactate threshold reflecting the point where lactate production exceeds lactate removal. Athletes with a higher lactate threshold can theoretically perform at higher average intensities without accumulating fatigue-inducing byproducts [34, 36].

Aerobic capacity testing under laboratory conditions may not be specific for intermittent sports such as soccer and is associated with certain limitations, including increased cost, the need for specialized equipment, increased time required for assessment, and limited accessibility. Hence, field testing has emerged as an attractive alternative, with the results of our systematic review demonstrating a higher preference for such assessment methods. Overall, the vast majority of the identified aerobic fitness tests were field based (26 vs. nine studies), which may be attributed to their reduced cost compared to laboratory testing, the ease of administration that allows for a large number of athletes to be assessed at once, and their practical relevance [30]. More specifically, field tests of different types were identified, such as continuous straight-line running (e.g. VAMEVAL test, Mognoni test, Conconi test), shuttle (e.g. 20-m multistage fitness test), intermittent shuttle (e.g. Yo-Yo intermittent tests, 30–15 IFT), and submaximal (e.g. submaximal warm-up test). Notably, intermittent tests are characterized by greater anaerobic demands compared to continuous running tests, with significantly higher blood lactate levels and faster end-test velocities [63]. Among the identified field tests, YYIR1 and YYIR2 were the most used. It is vital to recognize that the frequency of use does not necessarily equate to the superiority of a test, and that each protocol carries inherent strengths and limitations that must be considered before selection.

YYIR1 and YYIR2 tests involve running 2 × 20-m shuttles at increased speeds controlled by audio signals, interspersed with 10 s of active recovery. Consequently, their intermittent nature may better reflect the demands of the sport. The difference between these two versions lies in their initial speed (10 km/h in YYIR1 vs. 13 km/h in YYIR2) and the speed increases, which are more conservative in YYIR1 [64]. These differences pose distinct physiological demands, with YYIR2 presenting a greater blood lactate accumulation and anaerobic contribution [15, 42]. Hence, YYIR1 and YYIR2 should not be used interchangeably. Interestingly, the YYIR1 seems to be preferred in elite soccer players compared to YYIR2 (22 vs. ten studies). This preference could be attributed to its better suitability in evaluating aerobic adaptations due to the greater involvement of the aerobic system [64]. Rampinini et al. [15] demonstrated a strong correlation between YYIR1 and $\dot{V}$O_2max_ (*r* = 0.74, *p* < 0.05) in professional soccer players, while YYIR2 showed only moderate correlations with $\dot{V}$O_2max_ (*r* = 0.47, *p* < 0.05). However, Bangsbo et al. [64] reported a wide variability in YYIR1 performance among individuals with similar $\dot{V}$O_2max_ values. This suggests that YYIR1 performance reflects additional physiological qualities, such as recovery ability and anaerobic contribution, which are not captured by aerobic capacity alone. Therefore, YYIR1 and YYIR2 should not be used to estimate $\dot{V}$O_2max_, as they are not accurate measures of maximal aerobic capacity. Instead, their utility lies in assessing soccer-specific intermittent endurance, reflecting a player’s ability to sustain repeated high-intensity efforts. YYIR1 has been found to significantly differentiate between professionals and amateurs (2231 ± 294 vs. 1827 ± 292, *p* = 0.002, effect size [ES] = 1.14) [15], adult and youth (*p* < 0.05, ES = 1.21) [17], as well as male and female soccer players (2414 ± 456 vs. 1224 ± 255 m, *p* < 0.01) [16]. However, the YYIR1 and YYIR2 tests have limited suitability for training prescription of high-intensity interval training (HIIT), due to their distance-focused nature. This is not in alignment with the time-defined nature of HIIT sessions, which require the athlete to maintain a certain intensity over a prescribed time interval. Additionally, although Bok and Foster [30] demonstrated a high sensitivity to change for both YYIR1 (signal-to-noise ratio [SNR] = 2.7) and YYIR2 (SNR = 2.5), these values are considerably lower compared to the 30–15 IFT (SNR = 5.1).

The 30–15 IFT, which consists of 30-s shuttle runs interspersed with 15-s passive recovery periods and an increase in velocity by 0.5 km/h at the end of each 45-s stage, was used in six studies in our review. Notably, all of these studies were conducted within the last 7 years, potentially highlighting an emerging trend. In line with this, a recent survey of the current fitness testing practices of practitioners working in applied elite soccer settings showed that the 30–15 IFT was the most commonly used aerobic test [45]. The final velocity in km/h represents the main outcome variable, which reflects a combination of aerobic and anaerobic capacities, COD ability, and the inter-effort recovery ability, making it more appropriate for the prescription of short HIIT formats [65]. Other commonly used field tests employed in elite soccer included the YYIE2 (eight studies), the VAMEVAL test (eight studies), and the 20-m multistage fitness test (six studies). The VAMEVAL test has been reported to possess high criterion-related validity, with a correlation coefficient of *r* = 0.96 and a standard error of estimate of 2.8 mL/kg/min, rendering it a suitable field test for the evaluation of $\dot{V}$O_2max_ [30]. In addition, its continuous nature supports its use in the prescription of long format HIIT sessions, with the MAS representing the most frequently used outcome variable in systematic reviews. Similarly, the 20-m multistage fitness test exhibits criterion-related validity with a correlation coefficient of *r* = 0.85–0.91 and a standard error of estimate ranging from 3.5 to 5.9 mL/kg/min, depending on the population tested. While it is less precise than the VAMEVAL in $\dot{V}$O_2max_ estimation, its criterion-related validity is higher than the YYIR1, YYIR2, and 30–15 IFT for estimating $\dot{V}$O_2max_ in certain populations [30]. It should be noted though that many of these findings are largely dependent on the population assessed. However, we failed to find any studies examining criterion-related validity in elite soccer players.

The demanding training schedules and the fixture congestion encountered during the in-season phase in elite soccer environments pose significant challenges in the administration of aerobic fitness testing due to their fatiguing nature. Consequently, submaximal fitness tests have emerged as practical solutions, possibly owing to their short duration, non-disruptive and non-fatiguing nature [37]. This systematic review identified ten submaximal protocols, six of which were field based. Of particular advantage is that submaximal field protocols can be integrated into the training session as part of the warm-up, allowing a more frequent assessment of an individual’s training status. The submaximal YYIR1 [66], the submaximal YYIE2 [67], and the submaximal warm-up test [68] are viable short-duration (< 6 min) options, with the use of HR during exercise (HR_ex_) representing a reliable outcome variable (ICC 0.95–0.96, CV 1.4–1.6%). In addition, the HR_ex_ and the HR 1 min post-test from the submaximal warm-up test demonstrated significant large inverse relationships with the final velocity achieved in the 30–15 IFT (i.e. *r* = − 0.50 and *r* = − 0.76, respectively) [68].

Selecting the appropriate test is essential for accurate assessment and effective training prescription. To measure maximal aerobic capacity, laboratory testing remains the most accurate method. However, among field tests, the VAMEVAL is preferable due to the stronger criterion-related validity for $\dot{V}$O_2max_ compared to other options. While $\dot{V}$O_2max_ is a widely used measure, it represents only a limited aspect of a player’s working capacity and is less relevant to sports with predominant intermittent demands like soccer. If the goal is to assess a player's ability to perform repeated high-intensity efforts with brief recoveries, the Yo-Yo intermittent recovery (IRTs) and the 30–15 IFT represent the most suitable options. However, due to its enhanced prescriptive capabilities, the 30–15 IFT seems to be the most appropriate choice for evaluating soccer-specific endurance [69]. Ultimately, submaximal fitness testing allows for the ongoing evaluation of fitness and fatigue, facilitating informed adjustments to training loads as required.

#### RSA Assessment

The results of this systematic review revealed that RSA is being assessed to a considerably smaller extent than aerobic fitness in elite soccer. Logistical constraints, such as the time-consuming nature of RSA testing and the need for multiple staff members to ensure the adherence to the testing protocols, may limit its potential inclusion into a testing battery [12]. In particular, a wide variety of RSA tests were employed, including varying modes of exercise (overground sprinting vs. cycling), directions (linear vs. multidirectional), distances (20–40 m), repetitions (6–15), types of recovery (active vs. passive), and recovery durations (10–30 s), reflecting the complexity in RSA testing and the lack of a gold-standard testing protocol in elite soccer.

Linear RSA and RSA with CODs have been found to represent two separate attributes, as the shared variance between them is < 50% [23]. Although it could be assumed that the inclusion of CODs in RSA testing is of high relevance in sports where a vast number of directional changes occur, such as soccer [70, 71], linear RSA tests were the most frequently used modality. More specifically, 11 tests investigated linear RSA testing, whereas six tests assessed RSA involving at least one COD. Notably, the neuromuscular and metabolic systems are taxed in different ways, as the physiological load has been found to be higher in the presence of CODs in RSA testing due to higher accumulation of blood lactate [72]. In addition, the number and the angle of CODs can have a significant impact on the imposed neuromuscular load, with higher demands placed during sharper COD angles [73]. Consequently, the selection of an RSA test requires careful consideration of these aforementioned factors. In the context of the distinct demands of linear RSA and RSA with CODs, the RSA/repeated change of direction (RCOD) index, defined as the time of linear RSA divided by the time of RSA with CODs, was identified in this systematic review as a measure aiming to inform training focus in terms of linear RSA or RSA with CODs [23, 74, 75]. However, the practical application of the RSA/RCOD index may be limited due to the need to perform two different RSA tests, which is time consuming and potentially difficult to implement consistently in practice. In addition, the inherent limitations associated with its ratio-based nature may complicate its interpretation and reliability [76].

The recovery duration lasted between 10 and 30 s in the identified studies, with active recovery, mostly in the form of low-intensity jogging, being the type of recovery used in the majority of tests. The predominant use of active recovery may be attributed to its relevance to the soccer demands, where high-intensity bouts are interspersed with low-intensity activity [7, 39]. Active recovery has been shown to improve the rate of waste removal, due to enhanced blood flow, which can promote the buffering of hydrogen ions and the restoration of pH levels [77–79].

In terms of sprinting distances, the most used distances were 20 m, used by six tests, and 30 m, used by four tests, which may reflect the actual match-play sprint requirements, as it has been observed that the vast majority of sprint bouts during a soccer game are shorter than 30 m [7]. Although sprint repetitions ranged from 6 to 15, six repetitions were the most frequently used, identified in six different testing protocols. In instances where the total work is identical, the number of repetitions and sprint distances can influence the elicited physiological responses, as has been previously shown [60]. In particular, the ability to eliminate the decline in repeated sprint performance is more dependent on aerobic metabolism in a protocol with a higher number of repetitions and shorter distances (e.g. 12 × 20 m) compared to a protocol where a lower number of repetitions and longer distances are employed (e.g. 6 × 40 m).

The 6 × 40-m (20 + 20 m, 180° COD) shuttle sprint protocol with 20 s passive recovery was identified as the most commonly used RSA test in this systematic review, used in eight studies. RSA_best_, RSA_mean_, and performance decrement were the most frequently used outcome variables in this test. Despite its popularity, the use of passive recovery in this test may limit its ecological validity, given that low-intensity activities are performed during recovery periods in soccer matches [7]. Furthermore, the longer duration of each repetition, which is approximately 7 s, may introduce a greater reliance on anaerobic glycolysis, as opposed to the shorter duration sprint, where the energy requirements are being met by the phosphocreatine (PCr) system [20]. When considering all RSA test measures identified in this review, performance decrement (%), which is the ratio of RSA_mean_ to RSA_best_, was used in 14 tests (22 studies, 81%), RSA_total_ in 14 tests (14 studies, 52%), RSA_best_ in 13 tests (21 studies, 78%), and RSA_mean_ in ten tests (18 studies, 67%), and these represent the most frequent outcome variables used in RSA testing in elite soccer. Nevertheless, concerns about the reliability of performance decrement, which is an indicator of an individual’s ability to sustain sprint performance over successive sprints, have been previously reported in the literature [29, 41], and were in line with the results of the only study providing between-day reliability values on this measure in an elite soccer sample (ICC 0.17[90% CI: − 0.18 to 0.49], CV 30.2 [90% CI: 23.6–42.7]) [21].

It has been suggested that RSA testing protocols should align with the specific demands of soccer to enhance ecological validity [80, 81]. Recommendations from a recent systematic review [38] suggest that optimal RSA protocols should consist of seven sprints (to limit pacing strategies), involve distances of 30 m to engage top-speed ability, and incorporate active recovery periods that enable the achievement of work-to-rest ratios of 1:4 to 1:5, to effectively challenge the anaerobic energy system without complete PCr recovery between sprints. Based on these guidelines, the majority of the identified tests do not seem to align with these recommendations. The 7 × 30-m sprints with 25 s active recovery and the 7 × 30-m sprint completed on 20-s cycles represent the exception to this, which may suggest a more ecologically valid assessment. The introduction of a 180° turn in these protocols could further enhance their ecological validity by better replicating the demands of soccer.

In summary, RSA assessment seems to require a comprehensive understanding of the physiological responses elicited by different testing protocols. Selection of sprint distances, repetitions, the inclusion of CODs, their angle and number, and the type of recovery are all critical factors that should be considered due to their distinct neuromuscular and physiological requirements.

### Normative Values for Aerobic Fitness and RSA Tests

To our knowledge, this is the first systematic review collecting the normative values of common aerobic fitness and RSA tests in elite soccer players. In this way, in-depth insights into the aerobic fitness and RSA levels of high-performing soccer players can be acquired, enabling individual and group comparisons to be made, and measurable and realistic performance targets to be set. Furthermore, an understanding of the physical standards of elite performers can be valuable to the practitioners working with developmental soccer players, as it can lay the foundation for a structured long-term athletic development plan [82]. For a thorough investigation of the values reported from each study on $\dot{V}$O_2max_ from incremental treadmill testing, YYIR1 and YYIR2, VAMEVAL, YYIE2, 20-m multistage fitness test (beep test), 30–15 IFT, and the three most common RSA tests, readers are referred to Tables 5, 6, 7, and 8.

In terms of $\dot{V}$O_2max_, senior professionals (58.3 mL/kg/min) displayed a nearly identical median value to elite youth soccer players (57.5 mL/kg/min). However, caution should be exercised concerning these findings due to a smaller number of studies in elite youth populations. Unfortunately, none of the studies performed a direct comparison between these two groups within the same setting under similar testing conditions, which would have enhanced the comparability of the results. Signorelli et al. [59] compared younger (17–22 years old) and older (27–36 years old) Brazilian first division senior professionals, finding no significant differences between the two different groups (older group 63.2 ± 6.2 vs. younger group 62.7 ± 6.1). This could suggest that the high aerobic capacity is largely established early in a player’s professional career and remains stable with minimal improvement over time. The highest value identified in the elite youth group was 63.5 mL/kg/min, recorded in an Australian sample [83], whereas the highest value among senior professionals was 65.5 mL/kg/min, found in Spanish first division players [84]. In addition, eight studies in this review provided position-specific $\dot{V}$O_2max_ data [18, 62, 85–90]. Midfielders demonstrated the highest $\dot{V}$O_2max_ values, with a median of 59.7 mL/kg/min, which is indicative of the high aerobic levels required of midfield players in elite soccer. In contrast, defenders demonstrated the lowest $\dot{V}$O_2max_ levels, with a median of 56.4 mL/kg/min. Four studies further differentiated between centre-backs and full-backs, revealing median $\dot{V}$O_2max_ values of 54.8 mL/kg/min for centre-backs and 56.6 mL/kg/min for full-backs. Forwards exhibited a comparable median value of 56.8 mL/kg/min. These differences highlight the distinct aerobic demands of each position, which should be considered in programme design and the talent identification process.

In the YYIR1 test, senior professionals demonstrated a median distance of 2245 m (range 1348–2520) across 14 studies, which is similar to the elite youth group’s median distance of 2213 m (range 2054–3150), derived from six studies. Interestingly, the study reporting the lowest YYIR1 distance (1349 m) was from a high-level soccer club, where 80% of the players were representing their respective national teams [91]. This counterintuitive finding may be attributed to accumulated fatigue, as top-level players often participate in multiple competitions simultaneously, resulting in limited recovery times between matches. To facilitate a more accurate comparison between senior professionals and elite youth players while minimizing the influence of confounding variables (e.g. environmental conditions, differences in testing order, and timing of the day or season), we identified two studies that directly compared these groups. Both studies demonstrated a superior performance in senior professionals (2368 ± 265 m vs. 2054 ± 289 m [92] and 2414 ± 456 m vs. 2092 ± 260 m, *p* < 0.05 [16]). Similarly, the results for the YYIR2 distance were identical across the studies, for both senior professionals (median 958 m, range 613–1330) and the elite youth group (median 958 m, range 815–1147). However, when individual studies directly compared these two groups, a higher performance of senior professionals was observed, as shown by both Stevens et al. [93] (1300 ± 210 m vs. 1147 ± 244 m, ES = 0.67) and Krustrup et al. [42] (1050 vs. 815 m, *p* < 0.05). These findings suggest that although elite youth players exhibit high levels of aerobic ability, senior professionals have a greater high-intensity intermittent endurance ability, possibly due to increased number of years of exposure to high-intensity training and match-play. For the YYIE2 test, Bradley et al. [94] reported small, non-significant differences between Premier League (2364 ± 478 m), Championship (2268 ± 567 m), and League 1 senior players (2226 ± 432 m), suggesting comparable aerobic fitness levels across professional tiers. Elite youth players, on the other hand, outperformed non-elite counterparts across all playing positions in terms of YYIE2 distance, with effect sizes ranging from moderate to large (0.6–1.5) [95]. Finally, the single study comparing senior professionals and elite youth in the 30–15 IFT found slightly higher final velocities for elite youth players (19.5 ± 0.7 vs. 19.0 ± 1.0 km/h, ES = 0.39).

In terms of the 6 × 40-m (20 + 20 m) shuttle sprint protocol with 20 s passive recovery test, senior professionals outperformed elite youth soccer players, with a lower median value in terms of RSA_best_ (7.00 vs. 7.13 s) and RSA_mean_ (7.25 vs. 7.48 s). One study provided position-specific data, illustrating that full-backs displayed the best RSA performance (RSA_best_ 6.83 s; RSA_mean_ 7.18 s), whereas defenders had the worst (RSA_best_ 7.01 s; RSA_mean_ 7.40 s) [21]. This finding reflects the unique physical demands of the full-back position, owing their role in both offensive (e.g. overlapping runs) and defensive (e.g. recovery runs) situations within the match-play. Practitioners should consider these position-specific differences when designing RSA development training interventions.

Despite the practical utility of these normative values to drive evidence-informed training processes, certain limitations should be acknowledged in their interpretation and implementation. While aggregate data provide a broad overview, this approach may fail to capture nuanced methodological differences across studies. Consequently, variability in testing protocols and conditions may influence the comparability of the results and limit the generalization of these findings. For instance, the unequal number of studies in each group (e.g. 14 studies for senior professionals versus six studies for elite youth in YYIR1 test) may lead to a bias in the representativeness of the results and misleading conclusions. Factors such as different running surfaces [96], starting methods, distances behind the start line [97], and measurement equipment [98] have been shown to introduce substantial variability in the final outcome of a sprint test. These elements can have a major influence on the outcome measures derived from an RSA test, and as such, consideration of them is necessary. Similarly for $\dot{V}$O_2max_, the values reported by each study should be interpreted, and subsequently used, with caution, due to the variability in the employed protocols. Factors such as stage length, speed increments, percentage of inclination, and total duration can have significant implications on the final $\dot{V}$O_2max_ value [55]. More specifically, Astorino et al. [99] demonstrated that incremental treadmill protocols lasting approximately 7–10 min can optimize $\dot{V}$O_2max_ compared to longer protocols (> 13 min), which elicited a lower $\dot{V}$O_2max_ value. These factors highlight the need for universally standardized testing procedures to enhance the reliability and applicability of normative data. Until then, we recommend that practitioners exercise caution when comparing their results to different studies and ensure that any comparisons are made in context, by taking into account the specific testing conditions used.

### Reliability Data

The testing selection process should be influenced by the reliability or repeatability of a test [43, 100], as well as its sensitivity to detect small but important changes in performance [101]. Reliability represents a fundamental concept in the overall testing process, especially in high-performance sport where a limited amount of training adaptation is expected to occur [54]. A test with high variability fails to accurately represent an individual’s true performance, compromising the confidence in the precision of the results. It is important that the reliability levels of a test and its relevant outcome variables are determined within the specific cohort of interest, due to differences in the skill levels and training status of different populations [102, 103]. This systematic review found that a limited number of studies reported reliability data for aerobic fitness (*n* = 11) and RSA tests (*n* = 5), highlighting a gap in the literature, which may be attributable to the challenges associated with conducting test–retest procedures in elite environments.

The CV, representing the within-subject variation by dividing the standard deviation by the mean, was the most reported reliability metric for both aerobic fitness and RSA testing. A CV value of less than 10% is widely acknowledged as acceptable, yet this threshold may be arbitrary [104], as ‘highly’ variable outcome measures may be sensitive to change [105]. Consequently, a good understanding of the context at hand is required. The CV appears to be of great practical relevance to practice, as it can be used for the interpretation of performance changes [43]. In addition, the ICC represented the second most reported reliability metric for both physical qualities. The ICC refers to the between-subject variation (i.e. whether an individual maintains their ranking across repeated trials), and as such, is affected by group homogeneity. An ICC value of ≥ 0.75 is considered as ‘good’, whereas an ICC ≥ 0.90 is considered as ‘excellent’ [106]. Based on the generally accepted ICC and CV values, all the aerobic tests and outcome variables show good to excellent reliability, except the heart recovery at 30 s (ICC 0.58 [95% CI: 0.51–0.90], CV 19.5%) and 60 s (ICC 0.68 [95% CI: 0.56–0.93], CV 12.2%) after the completion of the submaximal YYIR1 [66]. The same was true for RSA tests, except for performance decrement in the 6 × 40-m (20 + 20 m) shuttle sprints with 25 s passive recovery (ICC 0.17 [90% CI: − 0.18 to 0.49], CV 30.2%) [21].

For aerobic testing, the 1000-m time trial demonstrated high reliability, with a low CV of 1.6% and an SEM of 2.86 s, making it a suitable tool for estimating MAS and prescribing aerobic conditioning intervals. In contrast, the YYIR2 test, while within acceptable CV range (4.2–9.6%) displayed higher variability compared to the 1000-m time trial and showed values similar to the YYIE2 (CV 3.9%). Despite the popularity of YYIR1 for assessing elite soccer players, we were unable to find a single study reporting reliability for the distance covered. Consequently, future research should focus on investigating the YYIR1 distance in elite soccer samples. Additionally, the Iintermittent endurance running (INTER) test, designed to replicate soccer-specific match demands by including shuttle runs, agility sprints, and straight-line sprints, demonstrated high reliability levels for both exercise tolerance time (ICC 0.75, CV 2.5%) and distance covered (ICC 0.79, CV 2.6%). For submaximal aerobic tests, the use of HR_ex_ seems to be well-justified for monitoring physiological adaptations in elite soccer players, considering the excellent reliability levels (ICC 0.95–0.96, CV 1.4–1.6%). On the other hand, the high variability of heart rate recovery (HRR) (ICC 0.58–0.93, CV 3.9–19.5%) may limit its usability as a reliable fitness indicator. When assessing RSA, practitioners are encouraged to prioritize metrics such as the RSA_total_ and RSA_mean_ over performance decrement measures, given the lack of accuracy and consistency of the latter.

Overall, it seems that a greater awareness needs to be adopted on the concept of reliability. A description of the procedures generating the reliability data is generally lacking, especially in relation to the between-day context (i.e. number of participants, days between the two assessments, etc.). It should be acknowledged that the test–retest process on different days (typically 3–7 days apart) captures the day-to-day fluctuations in an athlete's performance due to normal biological variation, which is paramount to accurately identifying true performance changes. In contexts requiring high precision, such as elite sports, stricter reliability benchmarks (e.g. CV ≤ 5%, ICC ≥ 0.90) may be more appropriate. However, there is currently no consensus on these stricter thresholds, and further research is needed to establish standardized guidelines. In addition, complementary tools such as Bland–Altman plots and analysis of variance (ANOVA) can provide information on the agreement and differences between trials that are not captured by ICC and CV, thus offering a more thorough understanding of a test's reliability. However, it is crucial to understand that the reliability determines the ‘noise’ of an outcome variable, and additional information on the typical variation over time (‘signal’) must be considered to draw meaningful conclusions on the utility of a test and its associated outcome variables. Ultimately, practitioners are recommended to establish their context-specific reliability measures, since the characteristics of each setting and athlete sample are distinct.

### Limitations and Directions for Future Research

Although this systematic review provided a comprehensive picture of aerobic fitness and RSA testing in elite soccer, certain limitations should be acknowledged. Firstly, it should be emphasized that the frequency of use of a fitness test in the literature should not be mistaken as an indicator of its effectiveness and its superiority. In addition, the terms ‘elite’ and ‘professional’ are often used interchangeably across the literature, leading to ambiguity and inconsistencies in participant classification. However, it is likely that these terms are used differently in different geographical regions and leagues. For example, the criteria for ‘elite’ performance in a top-tier European league may vary significantly from those in a smaller or developing soccer nation. This can be considered both as a limitation and a reflection of the existing soccer literature, highlighting the need for standardized terminology moving forward. While the Participant Classification Framework by McKay et al. [47] provides a solid foundation for categorizing athletes, it does not fully address the nuanced factors associated with soccer. Consequently, a soccer-specific classification system is needed to reflect the specifics of the sport’s hierarchical leagues structures, youth development pathways, and varying levels of professionalism across different regions. The variability in testing conditions, such as the different incremental test protocols with varying speeds and durations, as well as the different running surfaces in field-based testing, including natural and artificial turf, is an additional challenge in the establishment of universal normative standards, complicating the direct comparison of results across studies and limiting the generalizability of the findings. Lastly, due to the heterogeneity of testing methods identified in the present literature review, it was not possible to carry out a meta-analysis.

Areas requiring further investigation were identified in this systematic review. The development of a standardized process for assessing aerobic fitness and RSA would help to establish robust normative values and facilitate meaningful comparisons between different contexts. This standardization should not be limited to test selection and administration, but also include guidelines on data analysis, interpretation, and visualization to enhance overall consistency. In addition, the value of RSA testing has been questioned, due to the rare occurrences of repeated sprint sequences in a soccer match [107, 108]. As such, specific protocols evaluating repeated acceleration ability should be developed. Furthermore, greater emphasis on the concept of reliability is recommended, and a shift towards its consideration as an integral part of the reporting of results. Reliability studies using some of the most frequently identified tests such as YYIR1, 30–15 IFT, and 20-m multistage fitness test in elite soccer players should be performed. Longitudinal research should also aim to determine the sensitivity of these tests and their outcome variables. Combined with reliability data from the same athlete cohort, the signal-to-noise ratio can be quantified to provide an accurate evaluation of diagnostic ability and practical utility of each test. Such an approach will optimize the use of resources (e.g. time, staff, financial), reduce unnecessary testing, and produce quality data to inform decision making. Ultimately, this will support the establishment of fitness assessments tailored to the specific needs of elite soccer players.

## Conclusion

**Fig. 2:**
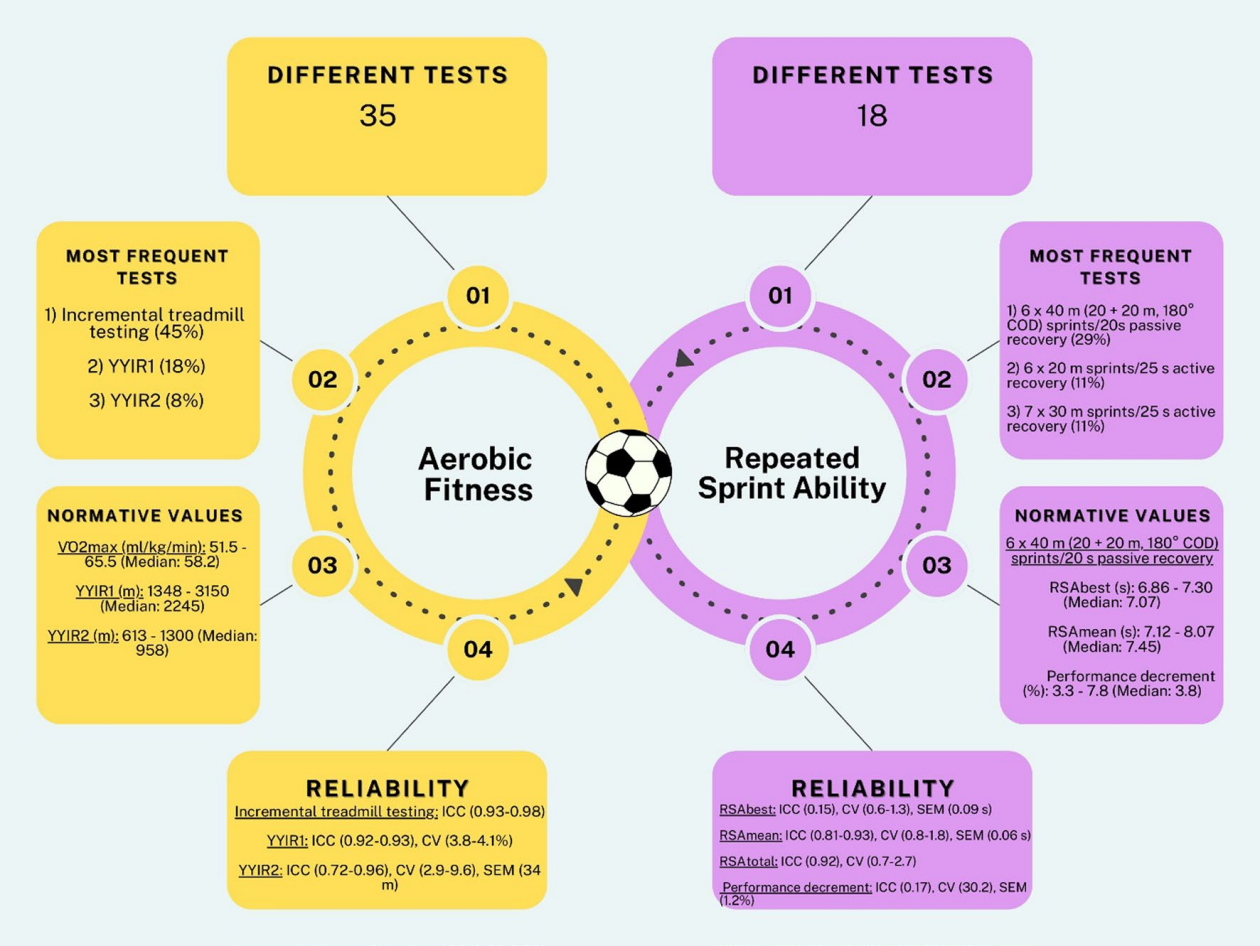
Aerobic fitness and RSA testing in elite male soccer. *COD* change of direction, *CV* coefficient of variation, *ICC* intraclass correlation coefficient, *RSA* repeated sprint ability, *RSA*_*best*_ best sprint time, *RSA*_*mean*_ mean sprint time, *RSA*_*total*_ total sprint time, *SEM* standard error of measurement, $\dot{V}$*O*_*2max*_ maximum oxygen uptake, *YYIR1* Yo-Yo intermittent recovery test level 1, *YYIR2* Yo-Yo intermittent recovery test level 2

The current systematic review provides a comprehensive overview of all tests and outcome variables used in elite male soccer to assess aerobic fitness and RSA (as shown in the infographic in Fig. 2), offering a valuable resource for researchers and practitioners. It should be noted that the frequency of use in the literature does not imply the superiority or effectiveness of these tests. The identification of 35 different aerobic fitness tests and 18 RSA tests illustrates the diverse methodologies employed in both research and practice, potentially influenced by differing testing philosophies, equipment accessibility, and logistical constraints. Field-based tests are predominantly utilized due to their practicality, cost-efficiency, and ability to assess multiple athletes simultaneously. The determination of a player’s $\dot{V}$O_2max_ using incremental treadmill testing represents the main choice for aerobic fitness testing, although significant variability across protocols was observed. A value of 58 mL/kg/min represents the median across studies, with only minimal differences between senior professionals and elite youth soccer players. Midfielders exhibited the highest $\dot{V}$O_2max_ values (59.7 mL/kg/min), while centre-backs presented the lowest (54.8 mL/kg/min). These normative values can serve as preliminary benchmarks for fitness evaluation and talent identification, yet caution is warranted due to methodological inconsistencies. YYIR1 and YYIR2 were also frequently used, potentially due to their historical precedence as the first field test enabling the assessment of soccer-specific intermittent endurance. Despite their widespread use, practitioners should acknowledge the limitations of these tests. An emerging trend towards the use of the 30–15 IFT and submaximal protocols has been identified, possibly due to the enhanced prescriptive ability and specificity of the former, and the minimal invasiveness of the latter, enabling its regular in-season use in elite applied soccer environments. For RSA assessment, the identified tests varied in terms of modes direction (linear vs. multidirectional), distances (20–40 m), repetitions (6–15), types of recovery (active vs. passive), and recovery durations (10–30 s). The 6 × 40-m (20 + 20 m, 180° COD) shuttle sprints with 20 s passive recovery test was the most commonly used RSA test, with performance decrement, RSA_total_, and RSA_mean_ being the main outcome variables used. Due to its low reliability, practitioners should avoid using the performance decrement. Additionally, the vast majority of the identified RSA tests failed to replicate the match-specific demands or to sufficiently challenge the relevant physiological systems, highlighting a gap between current testing practices and actual match-play requirements. Future research should prioritize determining the reliability and sensitivity (i.e. SNR) of the most common tests in elite soccer cohorts. Combined with their validity, this will enable a robust critical evaluation of these protocols, bridging the gaps between current practices and optimal fitness assessment.

## Supplementary Information

Below is the link to the electronic supplementary material.

Supplementary file1 (DOCX 260 KB)

## Declarations

### Data Availability Statement

The dataset generated and analysed during the current study are available from the corresponding author on reasonable request.
