# Supplementary material for: Assessment of Aerobic Fitness and Repeated Sprint Ability in Elite Male Soccer: A Systematic Review of Test Protocols Used in Practice and Research
Source: Sports Med. 2025 Apr 12;55(5):1233–64. doi: 10.1007/s40279-025-02188-4 (PMC12106594; doi:10.1007/s40279-025-02188-4)
Supplement: Supplementary file 1 — Supplementary file1 (DOCX 260 KB) [file 40279_2025_2188_MOESM1_ESM.docx]

**Title:** Assessment of Aerobic Fitness and Repeated Sprint Ability in Elite Male Soccer: A Systematic Review of Test Protocols used in Practice and Research

**Short title:** Aerobic Fitness and RSA Testing in Elite Male Soccer

**Authors:** Nikolaos D. Asimakidis^1,2^, Marco Beato^3^, Chris Bishop^1^, Anthony N. Turner^1^

^1^ Faculty of Science and Technology, London Sport Institute, Middlesex University, London, UK
^2^ Performance Department, Ipswich Town Football Club, Ipswich, UK

^3^ School of Health and Sports Sciences, University of Suffolk, Ipswich, UK

**Corresponding author**: Nikolaos D. Asimakidis

n.asimakidis@gmail.com

**Supplementary Table S1** Assessment scale used to evaluate the methodological quality of included articles [48]

| Question no. | Question |
| --- | --- |
|  | **Reporting** |
| 1. | Is the hypothesis/aim/objective of the study clearly described? |
| 2. | Are the main outcomes to be measured clearly described in the methods section? |
| 3. | Are the characteristics of the participants included in the study clearly described?  *Source defined, with characteristics included |
| 4. | Are the interventions/testing procedures in the study clearly described? |
| 5. | Are the main findings of the study clearly described? |
| 6. | Does the study provide estimates of the random variability in the data for main outcomes? |
| 7. | Have actual probability values been reported (e.g., 0.0035 rather than < 0.05) for the main outcomes except where the probability value is < 0.001?  *Exact correlation (r) and significance (p) values provided. The reporting of effect sizes (ESs) was deemed acceptable. |
|  | **External validity** |
| 8. | Were the subjects asked to participate in the study representative of the entire population from which they were recruited? |
|  | **Internal validity bias** |
| 9. | If any of the results of the study were based on “data dredging”, was this made clear? |
| 10. | Were the statistical tests used to assess the main outcomes appropriate? |
| 11. | Were the main outcome measures used accurate (reliable)? |

**Supplementary Table S2** Assessment of methodological quality [48]

| **Author** | **Question number** | | | | | | | | | | | **Total score** |
| --- | --- | --- | --- | --- | --- | --- | --- | --- | --- | --- | --- | --- |
|  | **1** | **2** | **3** | **4** | **6** | **7** | **10** | **11** | **16** | **18** | **20** |  |
| Unnithan et al. [109] | 1 | 1 | 1 | 1 | 1 | 1 | 1 | 0 | 1 | 1 | 1 | 10 |
| Wezenbeek et al. [110] | 1 | 0 | 1 | 1 | 1 | 1 | 1 | 0 | 1 | 1 | 1 | 9 |
| Guerra et al. [111] | 1 | 0 | 1 | 1 | 1 | 1 | 1 | 0 | 1 | 1 | 1 | 9 |
| Boraczyński et al. [112] | 1 | 1 | 1 | 1 | 1 | 1 | 1 | 0 | 1 | 1 | 1 | 10 |
| Manzi et al. [113] | 1 | 1 | 1 | 1 | 1 | 1 | 1 | 0 | 1 | 1 | 1 | 10 |
| Akyildiz et al. [114] | 1 | 0 | 1 | 1 | 0 | 1 | 1 | 0 | 0 | 1 | 1 | 7 |
| Schons et al. [115] | 1 | 1 | 1 | 1 | 1 | 1 | 0 | 0 | 1 | 1 | 1 | 9 |
| Enes et al. [116] | 1 | 1 | 1 | 1 | 1 | 1 | 1 | 0 | 1 | 1 | 1 | 10 |
| Colosio et al. [62] | 1 | 1 | 1 | 1 | 1 | 1 | 1 | 0 | 1 | 1 | 1 | 10 |
| Arregui-Martin et al. [117] | 1 | 0 | 1 | 0 | 1 | 1 | 1 | 0 | 1 | 1 | 1 | 8 |
| Azcárate et al. [118] | 1 | 1 | 1 | 0 | 1 | 1 | 1 | 9 | 1 | 1 | 1 | 9 |
| Querido and Clemente [119] | 1 | 1 | 1 | 1 | 1 | 1 | 1 | 0 | 1 | 1 | 1 | 10 |
| Michaelides et al. [120] | 1 | 0 | 1 | 0 | 1 | 1 | 1 | 0 | 1 | 1 | 1 | 8 |
| Lockie et al. [27] | 1 | 1 | 1 | 1 | 1 | 1 | 1 | 0 | 1 | 1 | 1 | 10 |
| Dolci et al. [83] | 1 | 1 | 1 | 1 | 1 | 1 | 1 | 0 | 1 | 1 | 1 | 10 |
| Silva et al. [121] | 1 | 1 | 1 | 1 | 1 | 0 | 0 | 0 | 1 | 1 | 1 | 8 |
| Martin et al. [122] | 1 | 1 | 1 | 1 | 1 | 1 | 1 | 0 | 1 | 1 | 1 | 10 |
| Parpa and Michaelides [85] | 1 | 1 | 1 | 0 | 1 | 1 | 1 | 0 | 1 | 1 | 1 | 9 |
| Saccà et al. [123] | 1 | 1 | 0 | 0 | 1 | 1 | 1 | 0 | 1 | 1 | 1 | 8 |
| Angoorani et al. [86] | 1 | 1 | 1 | 1 | 1 | 1 | 1 | 0 | 1 | 1 | 1 | 10 |
| Rabbani et al. [124] | 1 | 1 | 1 | 0 | 1 | 1 | 1 | 0 | 1 | 1 | 1 | 9 |
| Cardoso De Araújo et al. [125] | 1 | 1 | 1 | 1 | 1 | 1 | 1 | 0 | 1 | 1 | 0 | 9 |
| Rabbani et al. [126] | 1 | 1 | 1 | 1 | 1 | 1 | 1 | 0 | 1 | 1 | 1 | 10 |
| Jorge et al. [127] | 1 | 1 | 1 | 1 | 1 | 1 | 0 | 0 | 1 | 1 | 1 | 9 |
| Grazioli et al. [128] | 1 | 1 | 1 | 1 | 1 | 1 | 1 | 0 | 1 | 1 | 1 | 10 |
| Rago et al. [129] | 1 | 1 | 1 | 1 | 1 | 1 | 1 | 0 | 1 | 1 | 1 | 10 |
| Papadakis et al. [130] | 1 | 1 | 1 | 1 | 1 | 1 | 1 | 0 | 1 | 1 | 1 | 10 |
| Krespi et al. [131] | 1 | 0 | 0 | 1 | 1 | 1 | 1 | 0 | 1 | 1 | 1 | 8 |
| Radzimiński et al. [132] | 1 | 1 | 1 | 1 | 1 | 1 | 1 | 0 | 1 | 1 | 1 | 10 |
| Hoppe et al. [133] | 1 | 1 | 1 | 1 | 1 | 1 | 0 | 0 | 1 | 1 | 0 | 8 |
| Owen et al. [91] | 1 | 1 | 1 | 1 | 1 | 1 | 0 | 0 | 1 | 1 | 1 | 9 |
| Boraczyński et al. [134] | 1 | 1 | 1 | 1 | 1 | 1 | 1 | 0 | 1 | 1 | 1 | 10 |
| Bekris et al. [135] | 1 | 1 | 1 | 0 | 1 | 1 | 0 | 0 | 1 | 1 | 1 | 8 |
| Saidi et al. [136] | 1 | 1 | 1 | 1 | 1 | 1 | 1 | 0 | 1 | 1 | 0 | 9 |
| Clancy et al. [137] | 1 | 1 | 1 | 1 | 1 | 1 | 1 | 0 | 1 | 1 | 1 | 10 |
| Rabbani et al. [138] | 1 | 1 | 1 | 1 | 1 | 1 | 1 | 0 | 1 | 1 | 1 | 10 |
| Enright et al. [139] | 1 | 1 | 1 | 1 | 1 | 1 | 1 | 0 | 1 | 1 | 1 | 10 |
| Meckel et al. [140] | 1 | 1 | 1 | 1 | 1 | 1 | 0 | 0 | 1 | 1 | 1 | 9 |
| Malone et al. [13] | 1 | 0 | 1 | 1 | 1 | 1 | 1 | 0 | 1 | 1 | 1 | 9 |
| Rago et al. [141] | 1 | 1 | 1 | 0 | 1 | 1 | 1 | 0 | 1 | 1 | 1 | 9 |
| Los Arcos and Martins [142] | 1 | 1 | 1 | 1 | 1 | 1 | 1 | 0 | 1 | 1 | 1 | 10 |
| Almeida et al. [143] | 1 | 0 | 1 | 1 | 0 | 1 | 1 | 0 | 1 | 1 | 1 | 8 |
| Fessi et al. [144] | 1 | 1 | 1 | 1 | 1 | 1 | 0 | 0 | 1 | 1 | 1 | 9 |
| Castillo et al. [145] | 1 | 1 | 1 | 1 | 1 | 1 | 1 | 0 | 1 | 1 | 1 | 10 |
| Michaelides et al. [87] | 1 | 0 | 1 | 1 | 1 | 1 | 1 | 0 | 1 | 1 | 1 | 9 |
| Rodríguez-Fernández et al. [92] | 1 | 1 | 1 | 1 | 1 | 1 | 1 | 0 | 1 | 1 | 1 | 10 |
| Rabbani et al. [68] | 1 | 1 | 1 | 1 | 1 | 1 | 0 | 0 | 1 | 1 | 1 | 9 |
| Campos-Vazquez et al. [146] | 1 | 0 | 1 | 1 | 1 | 1 | 1 | 0 | 1 | 1 | 1 | 9 |
| Owen et al. [66] | 1 | 1 | 1 | 1 | 1 | 1 | 1 | 0 | 1 | 1 | 1 | 10 |
| Requena et al. [147] | 1 | 1 | 1 | 0 | 1 | 1 | 0 | 0 | 1 | 1 | 1 | 8 |
| Pareja-Blanco et al. [148] | 1 | 1 | 1 | 1 | 1 | 1 | 1 | 0 | 1 | 1 | 1 | 10 |
| Sapp et al. [149] | 1 | 0 | 1 | 1 | 1 | 0 | 0 | 0 | 0 | 1 | 1 | 6 |
| Yanci and Los Arcos [150] | 1 | 1 | 1 | 1 | 1 | 1 | 1 | 0 | 1 | 1 | 1 | 10 |
| Cerda-Kohler et al. [151] | 1 | 1 | 1 | 1 | 1 | 1 | 1 | 0 | 1 | 1 | 1 | 10 |
| Stevens et al. [93] | 1 | 1 | 1 | 1 | 1 | 1 | 1 | 0 | 1 | 1 | 1 | 10 |
| Fessi et al. [152] | 1 | 0 | 1 | 1 | 1 | 1 | 0 | 0 | 1 | 1 | 1 | 8 |
| Kobal et al. [17] | 1 | 1 | 1 | 1 | 1 | 1 | 1 | 0 | 1 | 1 | 1 | 10 |
| Spineti et al. [153] | 1 | 1 | 1 | 1 | 1 | 1 | 1 | 0 | 1 | 1 | 1 | 10 |
| Pareja-Blanco et al. [26] | 1 | 1 | 1 | 1 | 1 | 1 | 1 | 0 | 1 | 1 | 1 | 10 |
| Noon et al. [154] | 1 | 0 | 1 | 1 | 1 | 1 | 0 | 0 | 1 | 1 | 1 | 8 |
| Iaia et al. [155] | 1 | 1 | 1 | 1 | 1 | 1 | 1 | 0 | 1 | 1 | 1 | 10 |
| Wong et al. [75] | 1 | 1 | 1 | 1 | 1 | 1 | 0 | 0 | 1 | 1 | 1 | 9 |
| Arcos et al. [156] | 1 | 1 | 1 | 1 | 1 | 1 | 1 | 0 | 1 | 1 | 1 | 10 |
| Brocherie et al. [157] | 1 | 1 | 1 | 1 | 1 | 1 | 1 | 0 | 1 | 1 | 1 | 10 |
| Haddad et al. [158] | 1 | 1 | 1 | 1 | 1 | 1 | 1 | 0 | 1 | 1 | 1 | 10 |
| Wells et al. [159] | 1 | 0 | 1 | 0 | 1 | 1 | 1 | 0 | 1 | 1 | 1 | 8 |
| Manzi et al. [160] | 1 | 1 | 1 | 1 | 1 | 1 | 1 | 0 | 1 | 1 | 1 | 10 |
| Arcos et al. [161] | 1 | 1 | 1 | 1 | 1 | 1 | 1 | 0 | 1 | 1 | 1 | 10 |
| Koundourakis et al. [162] | 1 | 1 | 1 | 0 | 1 | 1 | 1 | 0 | 1 | 1 | 1 | 9 |
| Koundourakis et al. [163] | 1 | 1 | 1 | 1 | 1 | 1 | 1 | 0 | 1 | 1 | 1 | 10 |
| Ingebrigtsen et al. [25] | 1 | 1 | 1 | 1 | 1 | 1 | 1 | 0 | 1 | 1 | 1 | 10 |
| Manzi et al. [164] | 1 | 1 | 1 | 1 | 1 | 1 | 1 | 0 | 1 | 1 | 1 | 10 |
| Castagna et al. [165] | 1 | 1 | 1 | 1 | 1 | 1 | 1 | 0 | 1 | 1 | 1 | 10 |
| Bradley et al. [94] | 1 | 1 | 1 | 1 | 1 | 1 | 1 | 0 | 1 | 1 | 1 | 10 |
| Dellal and Wong [74] | 1 | 1 | 0 | 1 | 1 | 1 | 0 | 0 | 1 | 1 | 1 | 8 |
| Aandstad and Simon [166] | 1 | 1 | 1 | 1 | 1 | 1 | 1 | 0 | 1 | 1 | 1 | 10 |
| Rebelo et al. [95] | 1 | 1 | 1 | 1 | 1 | 1 | 1 | 0 | 1 | 1 | 1 | 10 |
| Silva et al. [167] | 1 | 1 | 1 | 1 | 1 | 1 | 1 | 0 | 1 | 1 | 1 | 10 |
| Tønnessen et al. [19] | 1 | 1 | 1 | 1 | 1 | 1 | 1 | 0 | 1 | 1 | 1 | 10 |
| Hoppe et al. [168] | 1 | 1 | 1 | 1 | 1 | 1 | 1 | 0 | 1 | 1 | 1 | 10 |
| Lago-Ballesteros [169] | 1 | 1 | 1 | 1 | 1 | 1 | 0 | 0 | 1 | 1 | 1 | 9 |
| Gibson et al. [170] | 1 | 1 | 1 | 1 | 1 | 1 | 0 | 0 | 1 | 1 | 1 | 9 |
| Boone et al. [88] | 1 | 1 | 1 | 1 | 1 | 1 | 1 | 0 | 1 | 1 | 1 | 10 |
| Owen et al. [171] | 1 | 1 | 1 | 1 | 1 | 1 | 1 | 0 | 1 | 1 | 1 | 10 |
| Akubat et al. [172] | 1 | 1 | 1 | 1 | 1 | 1 | 1 | 0 | 1 | 1 | 1 | 10 |
| Wong et al. [23] | 1 | 1 | 0 | 1 | 1 | 1 | 0 | 0 | 1 | 1 | 1 | 8 |
| Signorelli et al. [59] | 1 | 1 | 1 | 1 | 1 | 1 | 1 | 0 | 1 | 1 | 1 | 10 |
| Angius et al. [56] | 1 | 1 | 1 | 1 | 1 | 1 | 0 | 0 | 1 | 1 | 1 | 9 |
| Wells et al. [173] | 1 | 1 | 1 | 1 | 1 | 1 | 1 | 0 | 1 | 1 | 1 | 10 |
| Silva et al. [174] | 1 | 1 | 1 | 1 | 1 | 1 | 0 | 0 | 1 | 1 | 1 | 9 |
| Helgerud et al. [175] | 1 | 1 | 1 | 1 | 1 | 1 | 1 | 0 | 1 | 1 | 1 | 10 |
| Kalapotharakos et al. [176] | 1 | 1 | 1 | 1 | 1 | 1 | 1 | 0 | 1 | 1 | 1 | 10 |
| Bogdanis et al. [177] | 1 | 1 | 1 | 1 | 1 | 1 | 1 | 0 | 1 | 1 | 1 | 10 |
| Faude et al. [178] | 1 | 1 | 1 | 1 | 1 | 1 | 1 | 0 | 1 | 1 | 1 | 10 |
| Christensen et al. [179] | 1 | 1 | 1 | 1 | 1 | 1 | 0 | 0 | 1 | 1 | 1 | 9 |
| Ziogas et al. [34] | 1 | 1 | 1 | 1 | 1 | 1 | 1 | 0 | 1 | 1 | 1 | 10 |
| Bradley et al. [67] | 1 | 1 | 0 | 1 | 1 | 1 | 0 | 0 | 1 | 1 | 1 | 8 |
| Henderson et al. [180] | 1 | 0 | 1 | 0 | 1 | 1 | 0 | 0 | 1 | 1 | 1 | 7 |
| Chaouachi et al. [24] | 1 | 1 | 1 | 1 | 1 | 1 | 1 | 0 | 1 | 1 | 1 | 10 |
| Wong et al. [181] | 1 | 1 | 1 | 1 | 1 | 1 | 0 | 0 | 1 | 1 | 1 | 9 |
| López-Segovia et al. [182] | 1 | 0 | 1 | 1 | 1 | 1 | 1 | 0 | 1 | 1 | 1 | 9 |
| Rampinini et al. [15] | 1 | 1 | 0 | 1 | 1 | 1 | 1 | 0 | 1 | 1 | 1 | 9 |
| Rampinini et al. [22] | 1 | 1 | 0 | 1 | 1 | 1 | 1 | 0 | 1 | 1 | 1 | 9 |
| Mujika et al. [16] | 1 | 1 | 1 | 1 | 1 | 1 | 1 | 0 | 1 | 1 | 1 | 10 |
| Sotiropoulos et al. [183] | 1 | 1 | 1 | 1 | 1 | 1 | 0 | 0 | 1 | 1 | 1 | 9 |
| Sporis et al. [18] | 1 | 0 | 1 | 1 | 1 | 1 | 0 | 0 | 1 | 1 | 1 | 8 |
| Bravo et al. [184] | 1 | 1 | 1 | 1 | 1 | 1 | 1 | 0 | 1 | 1 | 1 | 10 |
| Clark et al. [185] | 1 | 1 | 1 | 1 | 1 | 1 | 1 | 0 | 1 | 1 | 1 | 10 |
| Aziz et al. [186] | 1 | 1 | 1 | 1 | 1 | 1 | 1 | 0 | 1 | 1 | 1 | 10 |
| Impellizzeri et al. [21] | 1 | 1 | 1 | 1 | 1 | 1 | 1 | 0 | 1 | 1 | 1 | 10 |
| Rampinini et al. [187] | 1 | 1 | 1 | 1 | 1 | 1 | 1 | 0 | 1 | 1 | 1 | 10 |
| Voutselas et al. [188] | 1 | 1 | 1 | 1 | 1 | 1 | 1 | 0 | 1 | 1 | 1 | 10 |
| Krustrup et al. [42] | 1 | 1 | 1 | 1 | 1 | 1 | 1 | 0 | 1 | 1 | 1 | 10 |
| Kalapotharakos et al. [189] | 1 | 1 | 1 | 1 | 1 | 1 | 0 | 0 | 1 | 1 | 1 | 9 |
| Chamari [190] | 1 | 0 | 1 | 1 | 1 | 1 | 0 | 0 | 1 | 1 | 1 | 8 |
| Guner et al. [61] | 1 | 1 | 1 | 1 | 1 | 1 | 0 | 0 | 1 | 1 | 1 | 9 |
| McMillan [191] | 1 | 1 | 1 | 1 | 1 | 1 | 1 | 0 | 1 | 1 | 1 | 10 |
| Chamari et al. [192] | 1 | 1 | 1 | 1 | 1 | 1 | 1 | 0 | 1 | 1 | 1 | 10 |
| Dupont et al. [193] | 1 | 1 | 1 | 1 | 1 | 1 | 1 | 0 | 1 | 1 | 1 | 10 |
| Ostojic [194] | 1 | 1 | 1 | 1 | 1 | 1 | 1 | 0 | 1 | 1 | 1 | 10 |
| Arnason et al. [195] | 1 | 1 | 1 | 1 | 1 | 1 | 1 | 0 | 1 | 1 | 1 | 10 |
| Lemmink et al. [196] | 1 | 1 | 1 | 1 | 1 | 1 | 0 | 0 | 1 | 1 | 1 | 9 |
| Edwards et al. [36] | 1 | 0 | 1 | 1 | 1 | 1 | 0 | 0 | 1 | 1 | 1 | 8 |
| Ozcakar [197] | 1 | 0 | 1 | 1 | 1 | 1 | 1 | 0 | 1 | 1 | 1 | 9 |
| Helgerud et al. [191] | 1 | 1 | 1 | 1 | 1 | 1 | 0 | 0 | 1 | 1 | 1 | 9 |
| Casajús [84] | 1 | 0 | 1 | 1 | 1 | 1 | 0 | 0 | 1 | 1 | 0 | 7 |
| Al-Hazzaa et al. [89] | 1 | 0 | 1 | 1 | 1 | 1 | 1 | 0 | 1 | 1 | 1 | 9 |
| Wisløff et al. [90] | 1 | 1 | 1 | 1 | 1 | 1 | 0 | 0 | 1 | 1 | 1 | 9 |
| Bangsbo and Lindquist [199] | 1 | 1 | 1 | 1 | 1 | 0 | 1 | 0 | 1 | 1 | 0 | 8 |
| Chin et al. [200] | 1 | 1 | 1 | 1 | 1 | 1 | 0 | 0 | 0 | 0 | 1 | 7 |
| Rhodes et al. [201] | 1 | 1 | 1 | 1 | 1 | 1 | 0 | 0 | 1 | 0 | 1 | 8 |

**Supplementary Table S3** Characteristics of the studies included in the review

| **Author** | **Sample size** | **Playing standard** | **Age (years)** | **Study design** | **Country (championship)** |
| --- | --- | --- | --- | --- | --- |
| Unnithan et al. [109] | 22 | Senior professionals | 25.2 ± 4.4 | Repeated measures | England (Championship) |
| Wezenbeek et al. [110] | 84 | Senior professionals | 24.7 ± 4.6 | Repeated measures | Belgium |
| Guerra et al. [111] | 24 | Senior professionals | 23.0 ± 3.9 | Intervention | Brazil (First Division) |
| Boraczyński et al. [112] | 25 | Senior professionals | 18.4 to 29.7 | Intervention | Poland (First Division) |
| Manzi et al. [113] | 62 | Senior professionals |  | Repeated measures | Italy (Serie A) |
| Akyildiz et al. [114] | 13 | Senior professionals | 21 ± 0.7 | Repeated measures | Turkey |
| Schons et al. [115] | 48 | Senior professionals | 22.8 ± 3.4 | Cross-sectional | Brazil (Professional competition of the state of Rio Grande do Sul) |
| Enes et al. [116] | 24 | Senior professionals | 26.7 ± 3.9 | Cross-sectional | Brazil (First Division) |
| Colosio et al. [62] | 186 | Senior professionals | 21.0 ± 4.5 | Cross-sectional | Belgium (First Division) |
| Arregui-Martin et al. [117] | 38 | Elite youth | 18.7 ± 1.1 | Repeated measures | Spain (First Division) |
| Azcárate et al. [118] | 20 | Senior professionals | 27.1 ± 3.1 | Repeated measures | Spain (Second Division) |
| Querido and Clemente [119] | 18 | Elite youth | 18.5 ± 0.4 | Repeated measures | Portugal (U19 First Division) |
| Michaelides et al. [120] | 19 | Senior professionals | 27.4 ± 3.7 | Repeated measures | Cyprus (First Division) |
| Lockie et al. [27] | 18 | Elite youth | 20.4 ± 1.5 | Cross-sectional | USA (Collegiate Division I) |
| Dolci et al. [83] | 11 | Elite youth | 18.5 ± 1.4 | Cross-sectional | Australia |
| Silva et al. [121] | 25 | Senior professionals | 28.1 ± 4.6 | Repeated measures | Qatar |
| Martin et al. [122] | 52 | Senior professionals | 25.0 ± 4.4 | Repeated measures | Italy (Serie A) & France (First Division) |
| Parpa and Michaelides [85] | 308 | Senior professionals | 25.4 ± 4.7 | Cross-sectional | Cyprus (First Division) |
| Saccà et al. [123] | 74 | Elite youth | 16 to 20 | Repeated measures | Italy (Primavera Championship) |
| Angoorani et al. [86] | 213 | Senior professionals | 25.0 ± 5.0 | Cross-sectional | Iran (First Division) |
| Rabbani et al. [124] | 22 | Senior professionals & elite youth | First team group: 28.3 ± 2.0 U19 group: 18.0 ± 0.4 | Cross sectional | Iran (First Division) |
| Cardoso De Araújo et al. [125] | 47 | Senior professionals | 17 to 34 | Cross-sectional | Germany (Bundesliga) |
| Rabbani et al. [126] | 14 | Senior professionals | 26.1 ± 5.7 | Repeated measures | Iran (First Division) |
| Jorge et al. [127] | 22 | Elite youth | 19.0 ± 0.8 | Repeated measures | Brazil (Sao Paulo’s Soccer Federation) |
| Grazioli et al. [128] | 23 | Senior professionals | 26.3 ± 5.6 | Repeated measures | Brazil |
| Rago et al. [129] | 17 | Senior professionals | 27.8 ± 3.9 | Repeated measures | Spain (First Division) |
| Papadakis et al. [130] | 16 | Senior professionals | 25.6 ± 3.2 | Repeated measures | Cyprus (First Division) |
| Krespi et al. [131] | 158 | Elite youth | 17.1 ± 0.79 | Intervention | Croatia |
| Radzimiński et al. [132] | 23 | Senior professionals | 27.9 ± 4.5 | Cross-sectional | Poland (First Division) |
| Hoppe et al. [133] | 54 | Elite youth | U21 group: 19.9 ± 0.3  U19 group: 17.6 ± 0.2 | Cross-sectional | Germany (Bundesliga youth academies) |
| Owen et al. [91] | 23 | Senior professionals | 25.3 ± 3.1 | Cross-sectional | Europe (UEFA Champions League) |
| Boraczyński et al. [134] | 25 | Senior professionals | 25.1 ± 4.5 | Cross-sectional | Poland (First Division) |
| Bekris et al. [135] | 24 | Senior professionals | 25.1 ± 4.5 | Repeated measures | Greece (First Division) |
| Saidi et al. [136] | 18 | Senior professionals | 20.1 ± 0.4 | Repeated measures | Tunisia (First Division) |
| Clancy et al. [137] | 16 | Elite youth | 19.2 ± 1.1 | Reliability | Scotland (Premier League) |
| Rabbani et al. [138] | 11 | Senior professionals | 27.2 ± 4.5 | Repeated measures | Iran (First Division) |
| Enright et al. [139] | 19 | Elite youth | 18.3 ± 0.2 | Reliability | England (Premier League) |
| Meckel et al. [140] | 18 | Senior professionals | 22-32 | Repeated measures | Israel (First Division) |
| Malone et al. [13] | 37 | Senior professionals | 25 ± 3 | Repeated measures | Portugal (First Division) |
| Rago et al. [141] | 14 | Senior professionals | 27.6 ± 3.0 | Cross-sectional | Italy (Serie B) |
| Los Arcos and Martins [142] | 97 | Elite youth | 20.5 ± 1.5 | Repeated measures | Spain (First Division) |
| Almeida et al. [143] | 40 | Senior professionals | 20.7 (18-34) | Repeated measures | Brazil |
| Fessi et al. [144] | 17 | Senior professionals | 26.1 ± 2.3 | Cross-sectional | Qatar (First Division) |
| Castillo et al. [145] | 162 | Elite youth | 21.3 ± 1.7 | Repeated measures | Spain (First Division) |
| Michaelides et al. [87] | 421 | Senior professionals | 25.0 ± 5.0 | Cross-sectional | Cyprus (First, Second & Third Division) |
| Rodríguez-Fernández et al. [92] | 33 | Senior professionals & elite youth | Professional group: 24.0 ± 2.8  Youth: 18.3 ± 0.8 | Repeated measures | Spain |
| Rabbani et al. [68] | 14 | Senior professionals | 26.7 ± 4.9 | Reliability & validity | Iran (First Division) |
| Campos-Vazquez et al. [146] | 12 | Senior professionals | 27.7 ± 4.3 | Repeated measures | Spain (Second Division) |
| Owen et al. [66] | 10 | Elite youth | 17.8 ± 0.5 | Reliability | England (Premier League) |
| Requena et al. [147] | 19 | Senior professionals | 26.2 ± 2.8 | Repeated measures | Spain (First Division) |
| Pareja-Blanco et al. [148] | 16 | Senior professionals | 23.8 ± 3.5 | Intervention | Spain |
| Sapp et al. [149] | 33 | Elite youth | 19.7 ± 1.2 | Repeated measures | USA (Collegiate Division I) |
| Yanci and Los Arcos [150] | 20 | Elite youth | 20.6 ± 1.8 | Repeated measures | Spain (First Division) |
| Cerda-Kohler et al. [151] | 17 | Senior professionals | 24.7 ± 3.7 | Cross-sectional | Chile (First Division) |
| Stevens et al. [93] | 63 | Senior professionals & elite youth | Senior group: 21 ± 3  Youth group: 17 ± 1 | Reliability & validity | Netherlands (First Division) |
| Fessi et al. [152] | 17 | Senior professionals | 23.7 ± 3.2 | Repeated measures | Qatar (First Division) |
| Kobal et al. [17] | 45 | Senior professionals & elite youth | Senior group: 22 ± 2.9  U20 group: 19 ± 0.6 | Cross-sectional | Brazil (Sao Paulo State First Division) |
| Spineti et al. [153] | 22 | Elite youth | 18.4 ± 0.4 | Intervention | Brazil (First Division) |
| Pareja-Blanco et al. [26] | 21 | Senior professionals | 24.3 ± 4.6 | Cross-sectional | Morocco |
| Noon et al. [154] | 14 | Elite youth | 17 ± 1 | Repeated measures | England (category 2 academy) |
| Iaia et al. [155] | 13 | Elite youth | 18.5 ± 1.0 | Intervention |  |
| Wong et al. [75] | 37 | Senior professionals & elite youth | First team group: 23.7 ± 5.4  U19 group: 17.4 ± 0.9 | Cross-sectional | Norway (First Division) |
| Arcos et al. [156] | 19 | Elite youth | 20.2 ± 1.9 | Repeated measures | Spain (First Division) |
| Brocherie et al. [157] | 16 | Senior professionals | 26.7 ± 4.0 | Cross-sectional | Qatar (National Team) |
| Haddad et al. [158] | 16 | Elite youth | 18.2 ± 1.2 | Intervention | Tunisia (First Division) |
| Wells et al. [159] | 16 | Senior professionals | 21.3 ± 2.1 | Intervention |  |
| Manzi et al. [160] | 17 | Senior professionals | 28.2 ± 2.2 | Repeated measures | Italy (Serie A) |
| Arcos et al. [161] | 22 | Senior professionals | 19.9 ± 1.7 | Intervention | Spain (Third Division) |
| Koundourakis et al. [162] | 67 | Senior professionals | 24.7 ± 1.0 | Repeated measures | Greece (First and Second Division) |
| Koundourakis et al. [163] | 55 | Senior professionals | 25.1 ± 5.1 | Repeated measures | Greece (First Division) |
| Ingebrigtsen et al. [25] | 57 | Senior professionals | 22.0 ± 5.0 | Cross-sectional | Norway (First, Second, and Third Division) |
| Manzi et al. [164] | 18 | Senior professionals | 28.4 ± 3.2 | Repeated measures | Italy (Serie A) |
| Castagna et al. [165] | 18 | Senior professionals | 28.6 ± 3.2 | Repeated measures | Italy (Serie A) |
| Bradley et al. [94] | 117 | Senior professionals | 26.0 ± 5.3 | Cross-sectional | England (Premier League, Championship, and League 1) |
| Dellal and Wong [74] | 26 | Senior professionals & elite youth |  | Cross-sectional | France (Ligue 1) |
| Aandstad and Simon [166] | 13 | Senior professionals | 23.0 ± 3.0 | Reliability & validity | Norway (Second Division) |
| Rebelo et al. [95] | 95 | Elite youth | 18.2 ± 0.6 | Cross-sectional | Portugal (First Division) |
| Silva et al. [167] | 13 | Senior professionals | 25.7 ± 4.6 | Repeated measures | Portugal (First Division) |
| Tønnessen et al. [19] | 872 | Senior professionals & elite youth | Men’s group: 24.3 ± 3.8  Youth group: 17.9 ± 1.2 | Cross-sectional | Norway (National Team, First and Second Division) |
| Hoppe et al. [168] | 11 | Senior professionals | 23.8 ± 3.0 | Cross-sectional | Germany (Third Division) |
| Lago-Ballesteros [169] | 42 | Senior professionals | 25 ± 5.2 | Repeated measures | Spain (First Division) |
| Gibson et al. [170] | 17 | Elite youth | 18.5 ± 0.7 | Cross-sectional | Scotland (First Division) |
| Boone et al. [88] | 289 | Senior professionals | 25.4 ± 4.9 | Cross-sectional | Belgium (First Division) |
| Owen et al. [171] | 15 | Senior professionals | 24.5 ± 3.4 | Intervention | Scotland (First Division) |
| Akubat et al. [172] | 9 | Elite youth | 17.0 ± 1.0 | Repeated measures | England (Football League Youth Alliance) |
| Wong et al. [23] | 18 | Senior professionals | 24.6 ± 5.5 | Cross-sectional | Hong Kong |
| Signorelli et al. [59] | 163 | Senior professionals | 25.0 ± 4.0 | Cross-sectional | Brazil (First Division) |
| Angius et al. [56] | 20 | Senior professionals | 24.1 ± 3.7 | Cross-sectional | Italy (Serie A) |
| Wells et al. [173] | 18 | Senior professionals | 23.2 ± 2.4 | Cross-sectional | England |
| Silva et al. [174] | 23 | Senior professionals | 25.7 ± 4.6 | Repeated measures | Portugal (First Division) |
| Helgerud et al. [175] | 21 | Senior professionals | 25 (20 to 31) | Intervention | Europe (UEFA Champions’ League) |
| Kalapotharakos et al. [176] | 12 | Senior professionals | 25.0 ± 5.0 | Repeated measures | Greece (First Division) |
| Bogdanis et al. [177] | 22 | Senior professionals | 22.3 ± 1.1 | Intervention | Greece |
| Faude et al. [178] | 15 | Senior professionals & elite youth | 19.5 ± 3.0 | Repeated measures | Germany (Third Division and Highest U19 National League) |
| Christensen et al. [179] | 18 | Senior professionals | 23.4 ± 3.5 | Intervention | Denmark (Third Division) |
| Ziogas et al. [34] | 129 | Senior professionals | 25.9 ± 4.9 | Cross-sectional | Greece (First, Second, and Third Division) |
| Bradley et al. [67] | 148 | Senior professionals |  | Cross-sectional | England & Denmark |
| Henderson et al. [180] | 36 | Senior professionals | 22.6 ± 5.2 | Cross-sectional | England (Premier League) |
| Chaouachi et al. [24] | 33 | Elite youth | 19.0 ± 1.0 | Cross-sectional | Tunisia (First Division) |
| Wong et al. [181] | 39 | Senior professionals | 22.8 ± 1.3 | Intervention | Hong Kong (First Division) |
| López-Segovia et al. [182] | 37 | Elite youth | 18.2 ± 0.7 | Repeated measures | Spain (First Division) |
| Rampinini et al. [15] | 13 | Senior professionals | 25.0 ± 4.0 | Cross-sectional | Italy |
| Rampinini et al. [22] | 12 | Senior professionals | 25.0 ± 4.0 | Cross-sectional | Italy (Serie C) |
| Mujika et al. [16] | 34 | Senior professionals & elite youth | Senior group: 23.8 ± 3.4  Junior group: 18.4 ± 0.9 | Cross-sectional | Spain (First Division) |
| Sotiropoulos et al. [183] | 58 | Senior professionals | 23.8 ± 2.7 | Intervention | Greece (First Division) |
| Sporis et al. [18] | 270 | Senior professionals | 28.3 ± 5.9 | Cross-sectional | Croatia (First Division) |
| Bravo et al. [184] | 22 | Elite youth | 17.3 ± 0.6 | Intervention |  |
| Clark et al. [185] | 42 | Senior professionals | 25 ± 3.5 | Repeated measures | England (Championship) |
| Aziz et al. [186] | 104 | Senior professionals | 24.1 ± 4.1 | Validity | Singapore (First Division) |
| Impellizzeri et al. [21] | 108 | Senior professionals | 22.0 ± 1.0 | Reliability & validity | Italy |
| Rampinini et al. [187] | 18 | Senior professionals | 26.2 ± 4.5 | Validity | Italy (Serie A) |
| Voutselas et al. [188] | 72 | Senior professionals | 25.1 ± 5.1 | Cross-sectional | Greece (First and Second Division) |
| Krustrup et al. [42] | 119 | Senior professionals & elite youth |  | Cross-sectional & reliability | Denmark (National team, First, and Second Division) |
| Kalapotharakos et al. [189] | 54 | Senior professionals | 24.3 ± 3.7 | Cross-sectional | Greece (First Division) |
| Chamari [190] | 24 | Senior professionals | 24.0 ± 2.0 | Cross-sectional | Tunisia (National Team) |
| Guner et al. [61] | 197 | Senior professionals | 24.6 ± 3.9 | Cross-sectional | Turkey (First Division) |
| McMillan [191] | 9 | Elite youth | 17.8 ± 0.2 | Cross-sectional | Scotland (First Division) |
| Chamari et al. [192] | 34 | Elite youth | 17.5 ± 1.1 | Cross-sectional | Tunisia (junior national team & First Division) |
| Dupont et al. [193] | 22 | Senior professionals | 20.2 ± 0.7 | Intervention | France |
| Ostojic [194] | 30 | Senior professionals | 24.0 ± 2.5 | Cross-sectional | Serbia (First Division) |
| Arnason et al. [195] | 306 | Senior professionals | 24 (16 to 38) | Cross-sectional | Iceland (First and Second Division) |
| Lemmink et al. [196] | 24 | Senior professionals | 23.0 ± 4.2 | Cross-sectional | Netherlands (First Division) |
| Edwards et al. [36] | 12 | Senior professionals | 26.2 ± 3.3 | Repeated measures |  |
| Ozcakar [197] | 29 | Senior professionals | 23.6 ± 3.6 | Cross-sectional | Turkey (First Division) |
| Helgerud et al. [198] | 19 | Elite youth | 18.1 ± 0.8 | Intervention | Norway (First Division) |
| Casajús [84] | 15 | Senior professionals | 25.8 ± 3.2 | Repeated measures | Spain (First Division) |
| Al-Hazzaa et al. [89] | 23 | Senior professionals | 25.2 ± 2.3 | Cross-sectional | Saudi Arabia (national team) |
| Wisløff et al. [90] | 29 | Senior professionals | 23.8 ± 3.8 | Cross-sectional | Norway (First Division) |
| Bangsbo and Lindquist [199] | 20 | Senior professionals | 23.9 ± 1.2 | Cross-sectional | Denmark (First Division) |
| Chin et al. [200] | 24 | Senior professionals | 26.3 ± 4.2 | Cross-sectional | Hong Kong (First Division) |
| Rhodes et al. [201] | 16 | Elite youth | 20.1 ± 1.1 | Cross-sectional | Canada (national olympic team) |

**Supplementary Table S4** Reliability data for aerobic fitness tests and outcome variables

| **Study** | **Test** | **Outcome variable** | **Reliability type** | **Reliability metric** | **Value** |
| --- | --- | --- | --- | --- | --- |
| Enright et al. [139] | Yo-Yo intermittent recovery test level 2 | Distance (m) | Interday | ICC | 0.96 (0.91-0.98) |
|  |  |  |  | CV | 4.2 (3.2-5.9) |
|  |  |  |  | SEM | 34.0 (26.5-48.1) |
|  |  |  |  | MDC | 94.1 |
| Boraczyński et al. [134] | 20 m multistage fitness test (beep test) | Distance (m) | Interday | ICC | 0.87 |
| Clancy et al. [137] | 1000 m time trial | Time to completion (s) | Interday | ICC | 0.82 (0.51-0.94) |
|  |  |  |  | SEM | 2.86 (2.08-4.71) |
|  |  |  |  | CV | 1.06 (0.68-1.44) |
|  |  |  |  | MDC | 4.56 |
| Rabbani et al. [68] | Submaximal warm-up test | HR exercise (b/min) | Interday | ICC | 0.95 (0.86–0.98) |
|  |  |  |  | CV | 1.4 (1.0–2.4) |
|  |  | HR recovery 60 seconds (b/min) |  | ICC | 0.84 (0.56–0.95) |
|  |  |  |  | CV | 7.0 (5.2–12.3) |
|  |  | HR post 1 minute (b/min) |  | ICC | 0.90 (0.71–0.97) |
|  |  |  |  | CV | 2.8 (2.1–4.9) |
| Owen et al. [66] | Submaximal Yo-Yo intermittent recovery test level 1 | HR at 6 minutes (completion of test) (b/min) | Interday | ICC | 0.96 (0.85 – 0.99) |
|  |  |  |  | CV | 1.6 |
|  |  |  |  | SEM | 1.9 |
|  |  |  |  | MDC | 5.4 |
|  |  | HR at 30s after completion (b/min) |  | ICC | 0.89 (0.55 – 0.98) |
|  |  |  |  | CV | 3.9 |
|  |  |  |  | SEM | 3.5 |
|  |  |  |  | MDC | 9.7 |
|  |  | HR at 60s after completion (b/min) |  | ICC | 0.81 (0.18 – 0.96) |
|  |  |  |  | CV | 5.6 |
|  |  |  |  | SEM | 4.4 |
|  |  |  |  | MDC | 12.3 |
|  |  | HR at 90s after completion (b/min) |  | ICC | 0.94 (0.76 – 0.99) |
|  |  |  |  | CV | 4.2 |
|  |  |  |  | SEM | 2.7 |
|  |  |  |  | MDC | 7.5 |
|  |  | HR at 120s after completion (b/min) |  | ICC | 0.93 (0.67 – 0.98) |
|  |  |  |  | CV | 4.4 |
|  |  |  |  | SEM | 2.7 |
|  |  |  |  | MDC | 7.5 |
|  |  | HRR at 30s after completion (%HRmax) |  | ICC | 0.58 (0.51 – 0.90) |
|  |  |  |  | CV | 19.5 |
|  |  |  |  | SEM | 1.9 |
|  |  |  |  | MDC | 4.3 |
|  |  | HRR at 60s after completion (%HRmax) |  | ICC | 0.68 (0.56 – 0.93) |
|  |  |  |  | CV | 12.2 |
|  |  |  |  | SEM | 2.5 |
|  |  |  |  | MDC | 7.0 |
|  |  | HRR at 90s after completion (%HRmax) |  | ICC | 0.90 (0.54 – 0.98) |
|  |  |  |  | CV | 5.9 |
|  |  |  |  | SEM | 1.7 |
|  |  |  |  | MDC | 4.6 |
|  |  | HRR at 120s after completion (%HRmax) |  | ICC | 0.93 (0.69 – 0.98) |
|  |  |  |  | CV | 5.7 |
|  |  |  |  | SEM | 1.7 |
|  |  |  |  | MDC | 4.6 |
| Ingebrigtsen et al. [25] | Yo-Yo intermittent recovery test level 1 | HR at 2nd minute of the test (b/min) | Interday | ICC | 0.92 |
|  |  |  |  | CV | 4.1 |
|  |  | HR at 4th minute of the test (b/min) |  | ICC | 0.93 |
|  |  |  |  | CV | 3.8 |
|  | Yo-Yo intermittent recovery test level 2 | HR at 2nd minute of the test (b/min) |  | ICC | 0.72 |
|  |  |  |  | CV | 2.9 |
| Aandstad and Simon [166] | Intermittent endurance running (INTER) soccer specific test | Time to exhaustion (s) | Interday | ICC | 0.75 (0.17- 0.94) |
|  |  |  |  | CV | 2.5 |
|  |  | Distance (m) |  | ICC | 0.79 (0.27- 0.96) |
|  |  |  |  | CV | 2.6 |
|  |  | Peak blood lactate concentration (mmol/L) |  | ICC | 0.95 (0.76-0.99) |
|  |  |  |  | CV | 4.8 |
|  |  | Mean blood lactate concentration (mmol/L) |  | ICC | 0.92 (0.65-0.98) |
|  |  |  |  | CV | 5.3 |
|  |  | HR_peak_ (b/min) |  | ICC | 0.98 (0.87-1.00) |
|  |  |  |  | CV | 0.6 |
|  |  | HR_mean_ for the final 15 s(b/min) |  | ICC | 0.95 (0.73- 0.99) |
|  |  |  |  | CV | 0.9 |
| Kalapotharakos et al. [176] | Incremental treadmill test to exhaustion | Velocity at 4 mmol/L (km/h) | Interday | ICC | 0.98 |
| Bogdanis et al. [177] | Incremental treadmill test to exhaustion | V̇O_2_max (ml/kg/min) | Interday | ICC | 0.94 |
|  |  | % V̇O_2_max at ventilatory threshold (%) |  | ICC | 0.93 |
|  | Yo-Yo intermittent endurance test level 2 | Distance (m) |  | ICC | 0.96 |
| Bradley et al. [67] | Yo-Yo intermittent endurance test level 2 | Distance (m) | Interday | CV | 3.9 |
|  | Submaximal Yo-Yo intermittent endurance test level 2 | HRmax (b/min) |  | CV | 1.4 |
| Krustrup et al. [42] | Yo-Yo intermittent recovery test level 2 | Distance (m) | Interday | CV | 9.6 |

*ICC* intraclass correlation coefficient; *CV* coefficient of variation; *SEM* standard error of measurement; *MDC* minimal detectable change; *MAS* maximal aerobic speed; V̇O_2_*max* maximum oxygen uptake; *HR* heart rate; *HRR* heart rate recovery; *HRpeak* peak heart rate; *HRmean* average heart rate; *HRmax* maximum heart rate

**Supplementary Table S5** Reliability data for RSA tests and outcome variables

| **Study** | **Test** | **Outcome variable** | **Reliability type** | **Reliability metric** | **Value** |
| --- | --- | --- | --- | --- | --- |
| Krespi et al. [131] | 6 x 2x15 m shuttle sprints with 20 seconds passive recovery | RSAmean (s) | Interday | ICC | 0.93 |
|  |  |  |  | CV | 1.8 |
| Christensen et al. [179] | 10 x 20 m sprints with 15 seconds active recovery | RSAbest (s) | Interday | CV | 0.6 |
|  |  | RSAtotal (s) |  | CV | 0.7 |
|  |  | Sprint fatigue index (%) |  | CV | 7.7 |
| Chaouachi et al. [24] | 7 x 30 m sprints with 25 seconds active recovery | RSAtotal (s) | Interday | ICC | 0.92 |
|  |  |  |  | CV | 2.7 |
| Wong et al. [23] | 6 x 20 m sprints with 25 seconds active recovery | RSAbest (s) | Interday | ICC | 0.88 |
|  |  |  |  | CV | 5.0 |
|  |  | RSAmean (s) |  | ICC | 0.90 |
|  |  |  |  | CV | 5.0 |
|  |  | RSAtotal (s) |  | ICC | 0.90 |
|  |  |  |  | CV | 5.0 |
|  |  | Performance decrement (%) |  | ICC | 0.11 |
|  |  |  |  | CV | 46 |
|  | 6 x 20 m sprints (with 4 changes of directions [100°] every 4m) with 25 seconds active recovery | RSAbest (s) |  | ICC | 0.79 |
|  |  |  |  | CV | 9.0 |
|  |  | RSAmean (s) |  | ICC | 0.80 |
|  |  |  |  | CV | 10.0 |
|  |  | RSAtotal (s) |  | ICC | 0.80 |
|  |  |  |  | CV | 10 |
|  |  | Performance decrement (%) |  | ICC | 0.17 |
|  |  |  |  | CV | 51 |
| Impellizzeri et al. [21] | 6 x 40 m (20+20) shuttle sprints with 20 seconds passive recovery | RSAbest (s) | Interday | ICC | 0.15 (-0.21 – 0.48) |
|  |  |  |  | CV | 1.3 (1.0-1.7) |
|  |  |  |  | SEM | 0.09 (0.07-0.12) |
|  |  | RSAmean (s) |  | ICC | 0.81 (0.64-0.90) |
|  |  |  |  | CV | 0.8 (0.6-1.0) |
|  |  |  |  | SEM | 0.06 (0.04-0.07) |
|  |  | Performance decrement (%) |  | ICC | 0.17 (-0.18 – 0.49) |
|  |  |  |  | CV | 30.2 (23.6-42.7) |
|  |  |  |  | SEM | 1.2 (0.9-1.6) |

*ICC* intraclass correlation coefficient; *CV* coefficient of variation; *SEM* standard error of measurement; *RSA* repeated sprint ability*; RSAbest* best sprint time; *RSAmean* mean sprint time; *RSAtotal* total sprint time
